# Summary-Based Methylome-Wide Association Analyses Suggest Potential Genetically Driven Epigenetic Heterogeneity of Alzheimer’s Disease

**DOI:** 10.3390/jcm9051489

**Published:** 2020-05-15

**Authors:** Alireza Nazarian, Anatoliy I. Yashin, Alexander M. Kulminski

**Affiliations:** Biodemography of Aging Research Unit, Social Science Research Institute, Duke University, Durham, NC 27705, USA; aiy@duke.edu

**Keywords:** neurodegenerative diseases, dementia, aging, GWAS, mQTLs, eQTLs, Alzheimer’s disease, Alzheimer’s disease pathogenesis, methylome-wide association analyses, summary data-based mendelian randomization

## Abstract

Alzheimer’s disease (AD) is a progressive neurodegenerative disorder with no curative treatment available. Exploring the genetic and non-genetic contributors to AD pathogenesis is essential to better understand its underlying biological mechanisms, and to develop novel preventive and therapeutic strategies. We investigated potential genetically driven epigenetic heterogeneity of AD through summary data-based Mendelian randomization (SMR), which combined results from our previous genome-wide association analyses with those from two publicly available methylation quantitative trait loci studies of blood and brain tissue samples. We found that 152 probes corresponding to 113 genes were epigenetically associated with AD at a Bonferroni-adjusted significance level of 5.49E-07. Of these, 10 genes had significant probes in both brain-specific and blood-based analyses. Comparing males vs. females and hypertensive vs. non-hypertensive subjects, we found that 22 and 79 probes had group-specific associations with AD, respectively, suggesting a potential role for such epigenetic modifications in the heterogeneous nature of AD. Our analyses provided stronger evidence for possible roles of four genes (i.e., *AIM2*, *C16orf80*, *DGUOK*, and *ST14*) in AD pathogenesis as they were also transcriptionally associated with AD. The identified associations suggest a list of prioritized genes for follow-up functional studies and advance our understanding of AD pathogenesis.

## 1. Introduction

Alzheimer’s disease (AD) is the major cause of dementia and is projected to affect more than 13 million people in the United States by 2050, thus imposing huge health and economic burdens [1,2]. Late onset AD is believed to be a multifactorial disease caused by complex interactions between various genetic and non-genetic factors [3]. Many genetic variants mapped to several chromosomal regions and genes have thus far been associated with AD by genome-wide association studies (GWAS) [4,5]; although, the vast majority of AD cases cannot be etiologically attributed to these variants [2,6]. Also, none of non-genetic AD-associated factors (e.g., age, cardiovascular risk factors, head trauma, depression, and educational attainment) has been proven to have a strong causal relationship with AD [7,8].

Epigenetic modifications of gene expression in interaction with non-genetic factors are hypothesized to contribute to AD development [6,9], particularly in light of the heterogeneous clinical manifestations of AD observed among patients with similar or identical genetic backgrounds [10]. The potential role of epigenetic mechanisms in AD pathogenesis has been widely investigated in cell lines, mouse models, post-mortem brain tissue, and blood cells [6,10,11,12,13]. Several studies have explored the global DNA methylation in AD cases compared with controls, although their findings have been inconclusive, with some reporting global hypomethylation in AD, some suggesting global hypermethylation in AD, and the others reporting no significant differences between cases and controls [12]. Previous studies have also provided many lines of evidence of associations between AD and gene-specific epigenetic modifications. They mainly investigated the DNA methylation and histone modification differences between AD cases and unaffected controls using candidate gene or genome-wide analysis approaches (e.g., pyrosequencing and array hybridization) which revealed AD-associated epigenetic modifications in some well-known AD genes, such as amyloid-β precursor protein (*APP*), Microtubule Associated Protein Tau (*MAPT*) [14], and Apolipoprotein E (*APOE*) [15], as well as in other genes [12]. For instance, Iwata et al. discovered CpG hypermethylation in *APP* and *MAPT* in post-mortem brain samples from AD patients, which were suggested to contribute to neural dysfunction and AD development [14]. Foraker et al. found that AD patients had a lower mean methylation level in 76 CpG sites across *APOE* gene compared with age-matched controls when hippocampus and frontal lobe samples were analyzed. However, *APOE* methylation was not statistically different between cases and controls in samples obtained from their cerebellum [15]. 

In most cases, epigenetically dysregulated genes were uniquely found in a single study [6,10,12,13], although AD-associated epigenetic modifications of some genes have been replicated in independent studies. For instance, several studies have reported CpG hypermethylation in the *ANK1* gene in different brain regions, such as entorhinal and prefrontal cortices, superior temporal gyrus, and/or hippocampus in AD patients [16,17,18]. Hypermethylated regions overlapping *DUSP22* gene were previously detected in entorhinal and dorsolateral prefrontal cortices and/or hippocampus of AD affected individuals [18,19], and CpG hypermethylation of *SORBS3* was detected in the cerebral cortex of AD patients and transgenic AD mouse models [11,20]. Moreover, differentially methylated regions overlapping *CDH23*, *RHBDF2*, and *RPL13* genes were reported in previous studies [16,17,21]. The mRNA expressions of these genes were also found to be altered in AD patients [16]. In addition, several genes whose associations with AD were replicated by independent GWAS [2], such as *ABCA7*, *BIN1*, *CLU*, *HLA-DRB5*, *SLC24A4*, and *SORL1*, are epigenetically implicated in AD as well [16,22,23]. The case-control studies and cell/animal models may not, however, reflect genetic contributions to AD-associated epigenetic modifications as they are more likely to identify the environmentally induced epigenetic alterations [6,9]. In addition to the studies using individual-level data, several epigenetically AD-associated genes, such as *BIN1*, *APOC1*, *HLA-DRB1*, *HLA-DRB5*, and *TOMM40*, have been reported by summary data-based analyses [24,25] which reflect genetically driven (i.e., through cis acting variants) epigenetic alterations [26].

In this study, we performed methylome-wide association (MWA) analyses of AD using the summary data-based Mendelian randomization (SMR) method [26] to investigate genetically driven epigenetic contributors to AD pathogenesis. Instead of analyzing individual-level data, the SMR method integrates the summary results from previous GWAS [27,28] and methylation quantitative trait loci (mQTLs) studies using blood samples [29] and brain tissue [30] in order to identify associations between AD and methylation alterations that may mediate the genetic associations examined by GWAS. Central to our study was to investigate potential genetically driven epigenetic heterogeneity of AD. Therefore, summary results from our previous GWAS which aimed to analyze genetic heterogeneity of AD in contrasting groups of subjects stratified based on their sex and history of hypertension (HTN) were used for our MWA analyses. Sex has been identified as a risk factor for AD and there are many reports highlighting sex disparities in epidemiological and clinical features of AD [31,32,33,34,35,36,37]. HTN is also a major cardiovascular risk factor for AD that may be involved in initiation and progression of the disease by causing structural and functional damages to cerebral microvasculature and promoting amyloid plaques formation [8,38,39]. By detecting several group-specific AD-associated single-nucleotide polymorphisms (SNPs) at P < 5E-06, our GWAS suggested that differences in the genetic architecture of AD between these contrasting groups may differentially contribute to AD pathogenesis [27,28]. Thus, the current study using summary results from these two GWAS may provide novel insights into potential genetically driven epigenetic heterogeneity of AD. To further validate significant findings, we compared our MWA results with those from our previous transcriptome-wide association (TWA) analyses of AD [27,28] that implemented the SMR method using the same GWAS summary results along with data from blood-based [40] and brain-specific [30,41] expression quantitative trait loci (eQTLs) studies. 

## 2. Methods

### 2.1. GWAS Data

This study makes use of the results of our previous genome-wide association meta-analyses [27,28]. Briefly, these meta-analyses were performed using genotype and phenotype data from four independent datasets: (1) Cardiovascular Health Study (CHS) [42]; (2) Framingham Heart Study (FHS) [43,44]; (3) Late-Onset Alzheimer’s Disease Family Study (LOADFS) from the National Institute on Aging [45], available to the research community through the dbGaP repository (https://www.ncbi.nlm.nih.gov/gap); and (4) Health and Retirement Study (HRS) [46], which can be accessed through dbGaP and the University of Michigan restricted access webpage (http://hrsonline.isr.umich.edu/index.php?p=data). These meta-analyses were performed under five analysis plans in which the genetic basis of AD was investigated among: (1) all subjects in each dataset, (2) only males, (3) only females [27], (4) only subjects with a history of HTN, or (5) only subjects with no history of HTN [28]. AD patients were mainly diagnosed clinically based on neurologic findings (e.g., using National Institute of Neurological and Communicative Disorders and Stroke-Alzheimer’s Disease and Related Disorders Association (NINCDS-ADRDA) criteria [47]) and were either identified directly (LOADFS and FHS datasets) or reported indirectly (CHS and HRS datasets) through the International Classification of Disease codes, Ninth revision (i.e., ICD-9:331.0 code). The numbers of AD cases were 2741, 952, 1789, 1262, and 796 under plans 1–5, respectively; and the numbers of unaffected controls were 14739, 6337, 8402, 9608, and 4010, respectively. The studied subjects were all of Caucasian ancestry to make samples more homogeneous.

For each analysis plan, the additive genetic associations of ~2 million SNPs with AD were investigated by fitting logistic regression (CHS and HRS cohorts with population-based design) [48] or generalized mixed logistic regression (LOADFS and FHS cohorts with family-based design) [49] models. The top five principal components of genotype data, birth year, and sex (except plans 2 and 3) of subjects were considered as fixed-effects covariates. In the case of LOADFS and FHS cohorts, family identifier was also included as a random-effects covariate in the fitted models to adjust for potential confounding from family structure. Individual GWAS results from the four datasets were then combined by inverse-variance meta-analysis [50]. Under plans 2–5 that aimed to investigate the genetic heterogeneity of AD through stratified analyses of datasets under consideration, group-specific SNPs effects were identified by a Wald chi-square test (df = 1) [51] which was performed for any SNPs with significant association signals in only one of the contrasting groups in order to determine whether the SNPs odds ratios were significantly different between males and females (plans 2 and 3) [27] and between hypertensive and non-hypertensive subjects (plans 4 and 5) [28].

(1) $$\chi^{2}=\frac{{\left(b_{1}-b_{2}\right)}^{2}}{se_{1}^{2}+se_{2}^{2}}$$

where *b*_1_ (*se*_1_) and *b*_2_ (*se*_2_) are the beta coefficients (and their standard errors) of a SNP in each of the two contrasting groups. 

### 2.2. mQTLs Data

The summary results from two previous mQTLs studies using blood samples (*n* = 1980) [29] and human brain tissue (*n* = 1160 from a meta-analysis of three independent brain-specific mQTLs data of mostly dorsolateral prefrontal cortex and fetal brain samples) [30] were also used for our analyses. The mQTLs studies provided genome-wide CpG methylation data using the Illumina Human Methylation 450 K array. The mQTLs data in the format compatible for MWA analyses can be downloaded at: https://cnsgenomics.com/software/smr/#DataResource. The annotation of probes was in accordance with the Illumina support files for Human Methylation 450K array. Probes which were located in the inter-genic regions (IGRs) (i.e., not located within any gene or within 1.5 kb of the transcription start site of any gene [52]) were annotated to their closest genes.

### 2.3. MWA Analysis

Under each of the five analysis plans, two sets of MWA analyses (i.e., blood-based and brain-specific) were performed by combining the results from our GWAS with publicly available summary results from the two mQTLs studies. MWA analyses were performed by the SMR package (v 0.710) [26] to identify SNPs that might be pleiotropically associated with AD and DNA methylation changes. The SMR package was run using default input arguments. Probes that had at least one significant mQTL (i.e., a SNP with P_mQTL_ < 5E-08) that was also among the SNPs in our GWAS were included. This resulted in the inclusion of sets of up to 90,357 and 90,848 probes with significant cis-mQTLs from blood-based and brain-specific mQTLs studies under the five analysis plans. 

Associations of any probes with AD were first sought through a SMR test, and significant associations were determined at a Bonferroni-adjusted significance level of 5.49E-07 (i.e., 0.05/91000) to account for multiple comparisons. Probes with significant P_SMR_ were then selected for heterogeneity in dependent instruments (HEIDI) testing to identify associations that were likely to arise from the pleiotropic effects of a single locus on both methylation changes and AD status (i.e., probes with P_HEIDI_ ≥ 0.05) and not from the linkage between adjacent variants that affected AD susceptibility and methylation patterns separately (i.e., probes with P_HEIDI_ < 0.05) [26]. Here, HRS was used as the reference panel for estimating pair-wise linkage disequilibrium and SNP clumping. 

To examine the consistency of probe effects in blood-based and brain-specific analyses, the b_SMR_ of any probes were compared between these analyses using the chi-square test mentioned above in the GWAS data section. In addition, probes that were detected in either males or females and in either hypertensive or non-hypertensive groups were subject to the chi-square test to find out whether their b_SMR_ were significantly different between the two contrasting groups (i.e., they had group-specific effects).

Finally, lists of AD-associated genes from MWA analyses were compared to those from our previous blood-based and brain-specific TWA analyses [27,28] to identify any overlaps between epigenetically and transcriptionally AD-associated genes.

### 2.4. Pathway Enrichment Analysis

Pathway enrichment analysis was performed to correlate nominally AD-associated genes in our MWA results with biological processes that might contribute to AD pathogenesis. Pathway-based analyses were performed by the GSA-SNP2 (i.e., gene set analysis-single nucleotide polymorphism2) package [53] using 1329 canonical pathways provided by the Broad Institute gene set enrichment analysis (GSEA) website [54] based on information from several pathway databases such as Kyoto Encyclopedia of Genes and Genomes (KEGG) [55], REACTOME pathway knowledgebase [56], Pathway Interaction Database (PID) [57], and Matrisome Project [58]. Significant AD-associated pathways were determined using plan-specific false discovery rates (FDR) [59] at which the numbers of possible false-positively detected pathways were smaller than 1 (i.e., FDR levels between 0.05 and 0.25).

### 2.5. Ethics Approval

This study focuses on secondary analysis of data obtained from dbGaP and the University of Michigan [42,43,44,45,46] (please see the Supporting Acknowledgment in Additional File 1) and does not involve gathering data from human subjects directly. The study was performed according to the Duke University Institutional Review Board (IRB) guidelines.

## 3. Results

### 3.1. Blood-Based MWA Analyses 

We found that 8, 31, 9, 6 and 84 probes passed both SMR at a Bonferroni-adjusted level of 5.49E-07 (P_SMR_ between 8.73E-20 and 5.26E-07) and HEIDI (P_HEIDI_ ≥ 0.05) tests under analysis plans 1–5, respectively (Additional File 1: Table S1). These probes were mapped to 5, 21, 9, 5, and 66 genes (71 chromosomal regions, i.e., cytogenetic bands, in total), respectively. Seventeen genes had more than one significant probe (2–9 probes per gene that were 51–61,765 base pairs apart and, in most cases, had the same top mQTLs). Top mQTLs corresponding to these probes were nominally significant (6.45E-06 ≤ P_GWAS_) in our genome-wide meta-analyses [27,28], except for the cg06750524 probe corresponding to the *APOE* gene whose top mQTLs had 2.15E-83 ≤ P_GWAS_ ≤ 8.19E-30 under the five analysis plans of interest. 

### 3.2. Brain-Specific MWA Analyses 

There were 2, 6, 4, 4, and 27 probes that passed both SMR at a Bonferroni-adjusted threshold of 5.49E-07 (P_SMR_ between 1.52E-12 and 5.17E-07) and HEIDI (P_HEIDI_ ≥ 0.05) tests under plans 1–5, respectively (Additional File 1: Table S2). These probes were mapped to 2, 5, 3, 4, and 24 genes (located in 26 chromosomal regions), respectively. Six genes had more than one significant probe (2–4 probes per gene that were 5–740 base pairs apart and were mostly influenced by the same genetic signal). Again, the top mQTLs corresponding to these probes were nominally significant (4.88E-05 ≤ P_GWAS_) in our GWAS except for the one corresponding to cg02613937 probe, which had 3.73E-63 ≤ P_GWAS_ ≤ 9.84E-24. This probe was mapped to the *TOMM40* gene, which is near the *APOE* gene.

### 3.3. Comparison of Blood-Based and Brain-Specific MWA Results

The consistency of blood-based and brain-specific results was examined by comparing the probes effect sizes and directions (i.e., the magnitudes and signs of b_SMR_) between the two analyses. The directions of effects were the same for ~77% of probes in both analyses and across five plans of interest. When the blood-based and brain-specific b_SMR_ were compared using a Wald chi-square test, less than 1% of probes (i.e., 0.006–0.073% across the five study plans) had significantly different effects at the Bonferroni-adjusted significance level. Probes corresponding to the following 10 genes were significantly associated with AD in both blood-based and brain-specific analyses (Table 1 and Table 2): *NANOS2* (plan 2), *HLA-DQB2* (plan 3), *FAM193B* (plan 4), *SLC6A7*, *BPGM*, *PSTK*, *KRTAP5-11*, *LECT1*, *ZNF598*, and *C16orf80* (plan 5). All but *BPGM* and *KRTAP5-11* had common probes in the two analyses, with directions of effects being the same and not significantly different at Bonferroni-adjusted level. The top mQTLs in blood-based and brain-specific analyses were the same for probes corresponding to *NANOS2*, *HLA-DQB2*, *FAM193B*, *SLC6A7*, *KRTAP5-11*, and *ZNF598*.

### 3.4. Group-Specific Findings

No probes/genes outside the *APOE* cluster genes region (i.e., chromosome 19q13.32) were significant in both males and females (i.e., plans 2 and 3). *LOC154449* (chromosome 6q27 region) was the only gene outside the *APOE* cluster genes region that had AD-associated probes in blood-based MWA analyses of both hypertensive and non-hypertensive subjects (i.e., plans 4 and 5). 

When the b_SMR_ of probes were compared using a Wald chi-square test, we found that 16 of 38 blood-based probes and six of eight brain-specific probes that were detected either in males or females had sex-specific effects at Bonferroni-adjusted significance levels of 0.00132 and 0.00625, respectively (Additional File 1: Tables S3 and S4). Among 88 and 29 blood-based and brain-specific probes that were detected in either hypertensive or non-hypertensive subjects, 58 and 21 probes had significantly different effects in the two groups at Bonferroni-adjusted significance levels of 0.00057 and 0.00172, respectively (Additional File 1: Tables S5 and S6).

### 3.5. Comparison of MWA and GWAS Results

To investigate the novelty of our findings with respect to their potential implication in AD pathogenesis, we determined whether there were AD-associated SNPs with significant P_GWAS_ at genome-wide (P_GWAS_ < 5E-08) or suggestive (5E-08 ≤ P_GWAS_ < 5E-06) significance levels within ±1 Mb regions and/or chromosomal regions of the detected probes in our genome-wide meta-analyses or in other studies reported by GRASP [4] and NHGRI-EBI GWAS [5] catalogs.

We identified AD-associated SNPs with P_GWAS_ < 5E-08 within ±1 Mb of probes corresponding to *APOE*, *TOMM40*, and *NANOS2* genes (all within the chromosome 19q13.32 region) in our genome-wide meta-analyses and previous GWAS [4,5]. No SNPs with P_GWAS_ < 5E-08 were found within ±1 Mb flanking regions of any other probes in our meta-analyses. However, AD-associated SNPs with P_GWAS_ < 5E-08 were previously reported by other studies within ±1 Mb of several other probes [4,5]. These probes were mapped to 22 genes (all outside the chromosome 19q13.32 region): *CLIC1*, *BRD2*, *HLA-DPB1*, *ITIH2*, *PHLDA1* (plan 2), *HLA-DQA2*, *HLA-DQB2*, *LECT1* (plan 3), and *SLC25A2*, *PPT2-EGFL8*, *EGFL8*, *COL11A2*, *TREM1*, *NDUFA4*, *ZNF394*, *CHRNA2*, *ITIH2*, *LECT1*, *CMIP*, *NGFR*, *LOC100288866*, *MUM1*, *SIGLEC12*, and *EBF4* (plan 5). 

In addition, the ±1 Mb flanking regions of several other probes attained 5E-08 ≤ P_GWAS_ < 5E-06 in our or previous GWAS. Detailed information about these probes/genes can be found in Additional File 1: Tables S1 and S2. For instance, there were AD-associated SNPs at suggestive significance levels within ±1 Mb of probes corresponding to *AP2A2*, *ADCY8*, *HLA-DQA2*, *HLA-DQB2*, and *SLC35C1* (all outside the chromosome 19q13.32 region) in our GWA meta-analyses.

### 3.6. Comparison of MWA and TWA Results

Analysis of overlaps between MWA and our previous TWA results [27,28] revealed that, among the potential epigenetically AD-associated genes, four genes also had significant AD-associated probes in TWA analyses (Table 3). These four genes, *AIM2*, *DGUOK*, *ST14*, and *C16orf80*, had significant probes in subjects with no history of HTN (i.e., plan 5). Of these genes, *C16orf80* had significant probes in both blood-based and brain-specific MWA analyses; *DGUOK* and *ST14* had AD-associated probes in blood-based analyses; and *AIM2* had significant probes in brain-specific analyses. With respect to the TWA analyses, *C16orf80* had significant probes in brain-specific analyses; *AIM2*, and *DGUOK* had significant probes in blood-based analyses; and *ST14* had AD-associated probes in TWA analyses of both blood samples and brain tissue. 

### 3.7. Pathway Enrichment Analyses

Pathway-based analyses (Table 4 and Table 5) revealed that AD-associated probes/genes from blood-based MWA analyses were enriched in 16 pathways (i.e., 7, 4, 4, and 3 pathways under plans 1, 2, 3, and 5, respectively). Of these, two pathways (i.e., *GABA-B* receptor activation (plans 1 and 3) and *GABA* receptor activation (plans 2 and 3)) were significant in more than one plan. We also found that nine pathways (i.e., 1, 2, 2, 3, and 3 significant pathways in plans 1–5, respectively) were associated with AD when brain-specific MWA results were enriched. Of these, two pathways (MHC class II antigen presentation (plans 1 and 3) and type II diabetes mellitus (plans 2 and 4)) were significant in more than one plan and were also enriched in both brain-specific and blood-based analyses. 

## 4. Discussion

Despite the detection of many genetic variants and identification of several non-genetic factors that may play roles in AD susceptibility, the definitive underlying mechanisms in most AD cases is unclear. Thus, epigenetic mechanisms may be key contributors to the heterogeneous nature of AD [9,10,13,23]. The epigenetic architecture of AD has been widely investigated in case-control studies and cell/animal models [12]. The AD-associated epigenetic modifications found in these studies can be environmentally induced or genetically driven (i.e., through cis acting variants). 

We combined the results from our previous GWAS [27,28] with data from two publicly available mQTLs studies of brain tissue [30] and blood samples [29] to identify genes that might be epigenetically associated with AD. In contrast to studies using individual-level data, epigenetic associations detected by summary data-based analyses are all genetically driven [26]. A major focus of our study was to explore potential genetically driven epigenetic heterogeneity of AD based on its two main risk factors (i.e., sex [31,32,33,34,35,36,37] and HTN [8,38,39]). Therefore, in order to investigate sex-specific and HTN-specific epigenetic changes, our MWA analyses were performed under five alternative plans in which summary results from GWAS on either all subjects, only males, only females [27], only subjects with a history of HTN, or only subjects with no history of HTN [28] were included in analyses. 

Our analyses demonstrated that 152 probes corresponding to 113 genes were epigenetically associated with AD. The top mQTLs corresponding to these probes were mostly nominally significant in our genome-wide meta-analyses. This might be in part due to suboptimal statistical power of our analyses which can be improved by analyzing larger datasets or more importantly due to the genetic heterogeneity of AD within and between the analyzed cohorts (i.e., LOADFS, CHS, FHS, and HRS). The ±1 Mb flanking regions of ~18% and ~34% of detected probes had attained P_GWAS_ < 5E-08 and 5E-08 ≤ P_GWAS_ < 5E-06, respectively, in our genome-wide meta-analyses or other studies reported by GWAS databases [4,5]. Comparing our findings with those detected in other SMR-based analyses of AD [24,25] revealed that *TOMM40*, which had significant probes in brain-specific analyses under all five plans of our study, was epigenetically associated with AD in a previous study [24].

Investigating group-specific epigenetic alterations, we found that probes corresponding to *APOE* and *TOMM40* genes (i.e., inside the chromosome 19q13.32 region) were significant in blood-based and brain-specific analyses, respectively, of both males and females (i.e., plans 2 and 3) and both hypertensive and non-hypertensive groups (i.e., plans 4 and 5). However, several probes (all outside the chromosome 19q13.32 region, except cg05206559 corresponding to *NANOS2* gene in males) were group-specifically associated with AD, indicating potential genetically driven epigenetic heterogeneity of AD based on the two studied risk factors. For instance, we found that among 38 and eight probes that were detected in blood-based and brain-specific analyses, respectively, in either males or females, 22 probes had sex-specific effects when their b_SMR_ were compared between the two sexes using a Wald chi-square test (Additional File 1: Tables S3 and S4). Comparing results from hypertensive and non-hypertensive groups, we found that there were 88 (blood-based analyses) and 29 (brain-specific analyses) significant probes outside the *APOE* region which were not in common between these two groups. Of these, 79 probes had group-specific effects when their b_SMR_ were compared between hypertensive and non-hypertensive groups (Additional File 1: Tables S5 and S6). Addressing genetic and epigenetic heterogeneities of AD is essential for understanding its pathogenesis and developing more efficient and personalized medical interventions tailored to the genetic and epigenetic profiles of individuals.

Our MWA analyses were performed using both brain-specific and blood-based mQTLs data which provided the opportunity to assess the consistency of potential AD-associated epigenetic changes detected in these analyses. Although the pattern of DNA methylation can be tissue- or cell-specific [6,60], previous studies have demonstrated the utility of blood samples for investigating AD-associated epigenetic modifications by reporting global or gene-specific methylation changes in AD subjects compared with matched healthy controls [61,62,63,64,65]. This might be due to the systemic sequelae of AD, as AD may extensively impact cellular and molecular processes in peripheral tissues and nonneural cells including red blood cells, leukocytes, and platelets [66,67,68,69,70,71]. In addition, blood-based analyses may provide more statistical power than brain-specific studies, which generally have smaller sample sizes due to difficulties in obtaining brain samples from living subjects. Consistent with previous reports, our findings supported the feasibility of using data from blood samples to investigate epigenetic changes involved in AD. The direction of blood-based and brain-specific effects were the same for ~77% of probes and the effects of less than 1% of probes were significantly different between the two analyses across the five analysis plans of interest. We also found that probes corresponding to 10 genes were associated with AD in both blood-based and brain-speficic MWA analyses (Table 1 and Table 2). Most of these genes were previously implicated in AD at genome-wide or suggestive significance levels by GWAS [4,5], except *SLC6A7*, *PSTK*, and *KRTAP5-11*. AD-associated SNPs at P_GWAS_ < 5E-08 were found within ±1 Mb of probes mapped to *NANOS2*, *HLA-DQB2*, and *LECT1* in our meta-analyses and/or previous GWAS. SNPs with 5E-08 ≤ P_GWAS_ < 5E-06 were found within ±1 Mb flanking regions of probes corresponding to *FAM193B*, *BPGM*, *ZNF598*, and *C16orf80*. Moreover, empirical evidence links some of these genes to AD in humans and animal models (e.g., *SLC6A7* [72] and *BPGM* [71]). 

It should be stressed that the identified AD-associated genes in summary-based analyses do not prove any definitive causal relationships. Instead, they suggest a list of prioritized genes whose potential roles in AD pathogenesis need to be validated by further functional studies [26]. In a recent study, Hannon et al. detected overlapping mQTL and eQTL signals with functional implications for several complex diseases/traits, such as Crohn’s disease, ulcerative colitis, blood lipids, height, and schizophrenia by comparing their SMR-based analyses [73]. Therefore, to further pinpoint potential targets, we compared the list of epigenetically AD-associated genes identified from MWA analyses with transcriptionally AD-associated genes identified from our previous TWA analyses [27,28]. 

Our comparisons identified a short list of four potentially AD-associated genes that had significant probes in both MWA and TWA analyses (i.e., *AIM2*, *DGUOK*, *ST14*, and *C16orf80* in non-hypertensive subjects with P_SMR_ between 4.62E-07 and 1.35E-10 in MWA analyses and between 2.18E-05 and 7.78E-07 in TWA analyses [28]). Probes corresponding to all genes but *AIM2* had group-specific effects when their b_SMR_ were compared between hypertensive and non-hypertensive groups using a Wald chi-square test (Additional File 1: Tables S5 and S6). AD-associated SNPs with P_GWAS_ < 5E-08 were not found within ±1 Mb flanking regions of these probes in our meta-analyses or other studies in GWAS databases [4,5], although several SNPs with 5E-08 ≤ P_GWAS_ < 5E-06 were previously reported within ±1 Mb of probes corresponding to *AIM2* [74] and *C16orf80* [75,76]. In addition, chromosomal regions corresponding to *ST14* [77] (i.e., 11q24.3 region) contained previously reported AD-associated SNPs at P < 5E-08.

A review of the literature provided additional insights, strengthening the potential roles of these four genes in AD. For instance, *AIM2* encodes a protein involved in regulating cell proliferation and innate immunity [78]. SNPs mapped to this gene were previously associated with white blood cells count at P_GWAS_ < 5E-08 [79]. *AIM2*, along with several other proteins, were suggested to initiate inflammasome formation in response to stimuli such as viruses, bacteria, and damaged cells. Inflammasomes mediate the release of pro-inflammatory cytokines, such as *IL-1β* and *IL-18*, that are believed to be involved in AD development [80,81,82]. *IL-1β* may increase in the blood, cerebrospinal fluid, and brain of AD patients and blood level of *IL-18* may increase in early stages of AD. *IL-1β* can activate astrocytes and microglia cells and stimulate the release of *APP* and amyloid-β (*Aβ*) from neurons. Also, *IL-18*, which is overexpressed in astrocytes, microglia, and neurons around *Aβ* plaques, may promote *Aβ* formation and mediate *tau* protein hyper-phosphorylation [82]. It was reported that methylene blue (MB), an inhibitor of inflammasome proteins such as *AIM2*, *NLRP3*, and *NLRC4* [80], can decelerate the production of *Aβ* plaques and neurofibrillary tangles. Thus, MB-based medications were suggested as potential treatments for AD [83]. Moreover, Wu et al. reported that *AIM2* knock-out mice exhibited behavioral changes and impaired auditory fear memory [84].

*DGUOK* encodes a mitochondrial enzyme involved in the purine metabolism pathway [78]. Mutations in this gene were linked to some mitochondrial disorders with Mendelian inheritance, such as mitochondrial depletion syndrome [85]. Mitochondrial dysfunction has also been reported as an important finding in neurons of AD patients [86,87]. Ansoleaga et al. showed that *DGUOK* was downregulated in the precuneus and entorhinal cortex of patients in AD stages III-IV and V-VI (Braak and Braak staging system [88]), respectively, compared with matched healthy controls [89]. In addition, SNPs mapped to *DGUOK* were associated with systemic lupus erythematosus at P_GWAS_ < 5E-08 [90]. The risks of developing AD and vascular dementia slightly increases among patients with autoimmune disorders, such as lupus erythematosus [91].

*ST14* encodes a membrane serine protease with tumor suppressor activity [78] that was not associated with AD or its risk factors at P_GWAS_ < 5E-06 by previous GWAS [4,5]. However, Wirz et al. found that the ortholog of *ST14* is overexpressed (i.e., 5.39-fold change with *p* < 0.008) in the frontal cortex of *APPswe/PS1dE9* transgenic mice harboring mutant forms of *APP* and *PSEN1* in response to *Aβ* plaque development [92]. Yin et al. reported that the mouse ortholog of *ST14* was upregulated in *Aβ* plaque-associated microglia cells in *5XFAD* transgenic mice harboring mutant forms of *APP* and *PSEN1* genes compared with aged-matched control mice [93]. 

*C16orf80* (also known as *BUG22* and *CFAP20*) encodes a highly conserved protein involved in the post-translational modification of *Tubulin* subunits of microtubules. Such modifications might be essential for microtubule function and stability in ciliated cells, such as sperm, and in neurons [94]. Microtubules are major component of neuronal transport machinery, in which defects can lead to neurodegenerative diseases (e.g., the role of microtubule-associated proteins, such as *tau* protein, in AD) [95,96]. In a previous study, Mendes Maia et al. reported that *Drosophila melanogaster* carrying mutant copies of the ortholog of *C16orf80* had a short lifespan and defects in body morphology, climbing activity, and locomotion, which were mostly reversed when gene expression was restored in the nervous system [94]. However, *C16orf80* was not previously associated with AD or its risk factors at P_GWAS_ < 5E-06 [4,5].

Our pathway enrichment analyses of the brain-specific and blood-based MWA results revealed that nine and 16 pathways were associated with AD, respectively. These pathways were mostly involved in biological processes such as immune system responses (e.g., MHC class II antigen presentation), mitochondrial function (e.g., TCA cycle and respiratory electron transport), neurogenesis, synaptic function, and neurotransmitter signaling (e.g., *L1CAM* interactions, *GABA* receptor activation, neurotransmitter receptors and postsynaptic signal transmission, and transmission across chemical synapses pathways) that have been implicated in AD pathogenesis [87,97,98,99,100,101,102,103]. Two enriched pathways (i.e., MHC class II antigen presentation and type II diabetes mellitus) were common between the brain-speficic and blood-based MWA analyses, highlighting potential links between AD and immune system responses [102,103] and type II diabetes mellitus as an important vascular risk factor for AD [104]. 

Despite its rigor, we acknowledge that this study has limitations that could be addressed by future research using different methodologies and data. Using summary results from GWAS with larger sample sizes is likely to increase the statistical power of analyses. However, it should be noted that increasing sample sizes may not necessarily result in considerably increased power of GWAS due to the genetic heterogeneity underlying complex diseases. As mentioned above, the summary-based methylome-/transcriptome-wide approaches cannot draw definitive causal relationships between the disease of interest and detected genes [26]. Such analyses can only help generate hypotheses regarding the possible involvement of a short list of genes in the pathogenesis of the studied disorder, which need to be validated empirically. Analyzing individual-level data which provide gene expressions and epigenetic profiles for the same case and control subjects would help obtaining a more definitive view of the underlying biological processes of AD and, in addition, may allow investigating the roles of non-genetic factors (e.g., smoking, medications that interfere with DNA methylation, exposure to metals, nutritional ingredients) in the observed transcriptome and epigenome changes. This is particularly important because epigenetic alterations can be environmentally induced [6,9]. It would also be interesting to investigate whether detected epigenome changes are associated with AD progression. This requires data from different AD stages [88] with sufficient sample sizes. The CHS, FHS, HRS, and LOADFS datasets analyzed in our study do not provide disease staging information for AD subjects. Finally, investigating cell-specific (i.e., neurons and different glial cells) epigenetic alterations may provide valuable additional insights into the epigenetic architecture of AD, although small sample sizes and insufficient statistical power can be a major problem for such studies. 

## 5. Conclusions 

Our MWA analyses revealed associations between AD and probes corresponding to 113 genes. Most of these genes were not associated with AD in previous GWAS and the ±1 Mb flanking regions of ~45% of detected probes did not attain P_GWAS_ < 5E-06 previously. The top mQTLs corresponding to these probes were mostly nominally significant in our GWAS which might be due to suboptimal sample sizes and statistical power of our analyses and/or the genetic heterogeneity of AD within and between the analyzed cohorts. Performing MWA analyses under five plans provided the opportunity to explore potential genetically driven epigenetic heterogeneity of AD in contrasting groups of subjects based on their sex and history of HTN. Comparing the MWA results from plans 2 and 3 (i.e., males vs. females) and from plans 4 and 5 (i.e., hypertensive vs. non-hypertensive subjects), we found that 22 and 79 probes were group-specifically associated with AD, respectively. Thus, this study suggests a role for genetically driven epigenetic modifications as contributing factors to the heterogeneous nature of AD, addressing of which may have translational impacts for implementing more efficient and personalized medical interventions (e.g., developing sex-specific therapeutic targets). The potential AD-genes associations detected here do not imply casualty and should only be used as a short list to prioritize candidate genes for future studies. The comparison of MWA and TWA results together with additional information from empirical studies strengthened the possible roles of four genes (i.e., *AIM2*, *C16orf80*, *DGUOK*, and *ST14*) in AD pathogenesis and helped further prioritize the list of potentially AD-associated genes for follow-up studies. Consistent with previous reports, our findings demonstrated the applicability of blood-based mQTLs data for the study of epigenetics mechanisms of AD as several genes and pathways were associated with AD in both brain-specific and blood-based MWA analyses and the probe effects detected in these analyses did not show significant differences. 

## Figures and Tables

**Table 1:** Blood-based methylome-wide association results for genes that had significant probes in both brain-specific and blood-based analyses.

ProbeID	Chr	ProbePos	Gene	SNP	Pos	A1	Freq	P_GWAS_	P_mQTL_	b_SMR_	SE_SMR_	P_SMR_	P_HEIDI_	N_HEIDI_	Current?	Previous?	Region?
**Plan 2: Only Males**																	
cg05206559	19q13.32	45913997	*NANOS2*	rs66529687	45914171	A	0.133	1.83E-04	2.84E-41	0.723	0.125	7.67E-09	3.28E-01	20	G	G	G
cg25673584	19q13.32	45914293	*NANOS2*	rs66529687	45914171	A	0.133	1.83E-04	4.40E-30	0.849	0.152	2.45E-08	1.23E-01	20	G	G	G
cg14192299	19q13.32	45914381	*NANOS2*	rs66529687	45914171	A	0.133	1.83E-04	6.71E-42	0.718	0.124	7.30E-09	1.08E-01	20	G	G	G
cg19702802	19q13.32	45914471	*NANOS2*	rs66529687	45914171	A	0.133	1.83E-04	3.22E-39	0.743	0.129	9.10E-09	1.03E-01	20	G	G	G
**Plan 3: Only Females**																	
cg10218546	6p21.32	32762046	*HLA-DQB2*	rs7768538	32762044	C	0.426	6.15E-05	1.30E-126	−0.304	0.060	3.27E-07	6.43E-02	20	S	G	G
**Plan 4: Hypertensive Subjects**																	
cg23395749	5q35.3	177557245	*FAM193B*	rs1001530	177558514	G	0.046	3.36E-04	2.55E-26	−0.484	0.088	3.08E-08	8.77E-02	5	N	S	S
**Plan 5: Non-hypertensive Subjects**																	
cg08631357	5q32	150209647	*SLC6A7*	rs10076748	150209303	A	0.107	1.77E-03	1.54E-193	0.288	0.056	3.18E-07	2.02E-01	20	N	N	G
cg23891049	7q33	134679117	*BPGM*	rs73441994	134679118	A	0.021	4.26E-02	1.18E-229	−0.156	0.030	1.70E-07	6.07E-01	4	N	S	S
cg24635736	10q26.13	122979534	*PSTK*	rs2421140	123027854	A	0.029	8.09E-03	2.67E-77	−0.346	0.060	6.12E-09	7.16E-01	8	N	N	N
cg05360847	11q13.4	71576873	*KRTAP5-11*	rs11827208	71578103	T	0.020	1.70E-03	3.47E-13	−0.942	0.159	3.50E-09	2.02E-01	4	N	N	S
cg17632299	13q14.3	52738831	*LECT1*	rs4885947	52735009	C	0.037	1.23E-03	7.51E-54	0.592	0.085	2.67E-12	1.34E-01	20	N	G	G
cg09557313	13q14.3	52739039	*LECT1*	rs4885947	52735009	C	0.037	1.23E-03	1.46E-40	0.675	0.100	1.37E-11	1.02E-01	20	N	G	G
cg09397293	16p13.3	2005032	*ZNF598*	rs72766639	2005819	A	0.174	1.69E-04	5.78E-51	0.688	0.116	3.06E-09	2.85E-01	20	N	S	G
cg26804891	16p13.3	2005241	*ZNF598*	rs11248905	1999727	T	0.181	4.88E-05	3.56E-98	0.539	0.080	1.62E-11	7.60E-02	20	N	S	G
cg08576185	16p13.3	2005683	*ZNF598*	rs72766639	2005819	A	0.174	1.69E-04	4.06E-44	0.740	0.126	4.76E-09	3.59E-01	20	N	S	G
cg10470208	16p13.3	2008700	*ZNF598*	rs1058474	1998795	T	0.181	6.82E-05	6.56E-19	1.112	0.209	1.02E-07	7.58E-02	14	N	S	G
cg06998361	16q21	58110599	*C16orf80*	rs10445026	58109349	G	0.069	5.00E-04	5.61E-97	−0.442	0.069	1.35E-10	2.53E-01	20	N	S	S

Genomic coordinates are based on Human Genome version 38 (hg38). Chr: chromosomal region (i.e., cytogenetic band); ProbePos: probe position; Gene: the gene or closest gene corresponding to the probe; SNP: top methylation quantitative trait locus (mQTL); Pos: SNP position; A1/Freq: SNP’s effect allele and its frequency; P_GWAS_: *p*-value of the SNP in genome-wide association meta-analysis; P_mQTL_: *p*-value of the SNP in mQTLs analysis; b_SMR_, SE_SMR_, and P_SMR_: beta coefficient, its standard error, and *p*-value of the probe in summary data-based Mendelian randomization (SMR) test; P_HEIDI_: *p*-value of the heterogeneity in dependent instruments (HEIDI) test; N_HEIDI_: number of single-nucleotide polymorphisms used for HEIDI test; Current?: whether there is any AD-associated SNP within ±1 Mb of the probe in the current genome-wide meta-analysis (N: None, G: SNP with P_GWAS_ < 5E-08, and S: SNP with 5E-08 ≤ P_GWAS_ < 5E-06); Previous?: whether there is any AD-associated SNP within ±1 Mb of the probe in previous GWAS (N: None, G: SNP with P_GWAS_ < 5E-08, and S: SNP with 5E-08 ≤ P_GWAS_ < 5E-06); Region?: whether there is any AD-associated SNP within the chromosomal region (i.e., cytogenetic band) corresponding to the probe (N: None, G: SNP with P_GWAS_ < 5E-08, and S: SNP with 5E-08 ≤ P_GWAS_ < 5E-06).

**Table 2:** Brain-specific methylome-wide association results for genes that had significant probes in both brain-specific and blood-based analyses.

ProbeID	Chr	ProbePos	Gene	SNP	Pos	A1	Freq	P_GWAS_	P_mQTL_	b_SMR_	SE_SMR_	P_SMR_	P_HEIDI_	N_HEIDI_	Current?	Previous?	Region?
**Plan 2: Only Males**																	
cg05206559	19q13.32	45913997	*NANOS2*	rs66529687	45914171	G	0.867	1.83E-04	5.86E-298	0.272	0.043	2.96E-10	8.50E-01	19	G	G	G
**Plan 3: Only Females**																	
cg04322111	6p21.32	32761987	*HLA-DQB2*	rs7768538	32762044	A	0.574	6.15E-05	0	−0.201	0.039	2.21E-07	8.61E-02	20	S	G	G
cg10218546	6p21.32	32762046	*HLA-DQB2*	rs7768538	32762044	A	0.574	6.15E-05	0	−0.198	0.038	2.18E-07	8.32E-02	20	S	G	G
**Plan 4: Hypertensive Subjects**																	
cg23395749	5q35.3	177557245	*FAM193B*	rs1001530	177558514	A	0.954	3.36E-04	2.34E-15	−0.791	0.157	5.17E-07	1.01E-01	5	N	S	S
**Plan 5: Non-hypertensive Subjects**																	
cg08631357	5q32	150209647	*SLC6A7*	rs10076748	150209303	C	0.893	1.77E-03	2.82E-295	0.230	0.045	2.76E-07	2.24E-01	18	N	N	G
cg10308629	7q33	134670051	*BPGM*	rs73439998	134663724	C	0.979	3.01E-02	9.28E-48	−0.520	0.101	2.88E-07	2.57E-01	3	N	S	S
cg24635736	10q26.13	122979534	*PSTK*	rs13328826	122992107	A	0.970	6.26E-03	2.48E-20	−0.374	0.072	1.68E-07	8.24E-01	3	N	N	N
cg15567360	11q13.4	71611653	*KRTAP5-11*	rs11827208	71578103	C	0.980	1.70E-03	9.66E-10	−0.679	0.130	1.67E-07	3.71E-01	3	N	N	S
cg09557313	13q14.3	52739039	*LECT1*	rs4885961	52755200	C	0.960	4.63E-03	6.93E-31	0.547	0.103	1.06E-07	5.67E-01	7	N	G	G
cg07011318	16p13.3	2004943	*ZNF598*	rs72766639	2005819	G	0.826	1.69E-04	0	0.291	0.046	1.96E-10	1.12E-01	17	N	S	G
cg09397293	16p13.3	2005032	*ZNF598*	rs72766639	2005819	G	0.826	1.69E-04	0	0.282	0.044	1.86E-10	1.13E-01	18	N	S	G
cg05211189	16p13.3	2005402	*ZNF598*	rs11542302	1986934	T	0.819	7.26E-05	0	0.283	0.043	7.47E-11	1.01E-01	18	N	S	G
cg08576185	16p13.3	2005683	*ZNF598*	rs72766639	2005819	G	0.826	1.69E-04	0	0.295	0.046	2.00E-10	9.02E-02	16	N	S	G
cg06998361	16q21	58110599	*C16orf80*	rs74019790	58107923	T	0.931	5.00E-04	4.77E-20	−0.591	0.109	5.49E-08	6.81E-01	11	N	S	S

Genomic coordinates are based on Human Genome version 38 (hg38). Chr: chromosomal region (i.e., cytogenetic band); ProbePos: probe position; Gene: the gene or closest gene corresponding to the probe; SNP: top methylation quantitative trait locus (mQTL); Pos: SNP position; A1/Freq: SNP’s effect allele and its frequency; P_GWAS_: *p*-value of the SNP in genome-wide association meta-analysis; P_mQTL_: *p*-value of the SNP in mQTLs analysis; b_SMR_, SE_SMR_, and P_SMR_: beta coefficient, its standard error, and *p*-value of the probe in summary data-based Mendelian randomization (SMR) test; P_HEIDI_: *p*-value of the heterogeneity in dependent instruments (HEIDI) test; N_HEIDI_: number of single-nucleotide polymorphisms used for HEIDI test; Current?: whether there is any AD-associated SNP within ±1 Mb of the probe in the current genome-wide meta-analysis (N: None, G: SNP with P_GWAS_ < 5E-08, and S: SNP with 5E-08 ≤ P_GWAS_ < 5E-06); Previous?: whether there is any AD-associated SNP within ±1 Mb of the probe in previous GWAS (N: None, G: SNP with P_GWAS_ < 5E-08, and S: SNP with 5E-08 ≤ P_GWAS_ < 5E-06); Region?: whether there is any AD-associated SNP within the chromosomal region (i.e., cytogenetic band) corresponding to the probe (N: None, G: SNP with P_GWAS_ < 5E-08, and S: SNP with 5E-08 ≤ P_GWAS_ < 5E-06).

**Table 3:** Methylome-wide association results for the four genes that had epigenetically and transcriptionally AD-associated probes.

ProbeID	Chr	ProbePos	Gene	SNP	Pos	A1	Freq	P_GWAS_	P_mQTL_	b_SMR_	SE_SMR_	P_SMR_	P_HEIDI_	N_HEIDI_	Current?	Previous?	Region?
**Blood-based Analyses**																	
cg03063511	2p13.1	73930386	*DGUOK*	rs6737156	73932607	C	0.036	5.62E-03	2.71E-227	−0.247	0.041	2.74E-09	1.09E-01	11	N	N	N
cg02850715	11q24.3	130159317	*ST14*	rs34008994	130165703	T	0.096	1.55E-04	1.21E-26	−0.812	0.138	4.14E-09	7.87E-01	20	N	N	G
cg21029769	11q24.3	130159620	*ST14*	rs34008994	130165703	T	0.096	1.55E-04	4.09E-18	−1.006	0.184	4.58E-08	9.16E-01	20	N	N	G
cg06998361	16q21	58110599	*C16orf80*	rs10445026	58109349	G	0.069	5.00E-04	5.61E-97	−0.442	0.069	1.35E-10	2.53E-01	20	N	S	S
**Brain-specific Analyses**																	
cg11003133	1q23.1	159076601	*AIM2*	rs16841642	159077008	G	0.952	5.30E-03	6.30E-82	−0.312	0.062	4.62E-07	3.40E-01	18	N	S	N
cg06998361	16q21	58110599	*C16orf80*	rs74019790	58107923	T	0.931	5.00E-04	4.77E-20	−0.591	0.109	5.49E-08	6.81E-01	11	N	S	S

Genomic coordinates are based on Human Genome version 38 (hg38). Chr: chromosomal region (i.e., cytogenetic band); ProbePos: probe position; Gene: the gene or closest gene corresponding to the probe; SNP: top methylation quantitative trait locus (mQTL); Pos: SNP position; A1/Freq: SNP’s effect allele and its frequency; PGWAS: *p*-value of the SNP in genome-wide association meta-analysis; PmQTL: *p*-value of the SNP in mQTLs analysis; bSMR, SESMR, and PSMR: beta coefficient, its standard error, and *p*-value of the probe in summary data-based Mendelian randomization (SMR) test; PHEIDI: *p*-value of the heterogeneity in dependent instruments (HEIDI) test; NHEIDI: number of single-nucleotide polymorphisms used for HEIDI test; Current?: whether there is any AD-associated SNP within ±1 Mb of the probe in the current genome-wide meta-analysis (N: None, G: SNP with PGWAS < 5E-08, and S: SNP with 5E-08 ≤ PGWAS < 5E-06); Previous?: whether there is any AD-associated SNP within ±1 Mb of the probe in previous GWAS (N: None, G: SNP with PGWAS < 5E-08, and S: SNP with 5E-08 ≤ PGWAS < 5E-06); Region?: whether there is any AD-associated SNP within the chromosomal region (i.e., cytogenetic band) corresponding to the probe (N: None, G: SNP with PGWAS < 5E-08, and S: SNP with 5E-08 ≤ PGWAS < 5E-06).

**Table 4:** Pathway-enrichment of blood-based methylome-wide association results.

Pathway	Pathway Source	GSEA ID	Size	Count	Z-Score	*p*-Value	*q*-Value
**Plan 1: All Subjects**							
Type II diabetes mellitus	KEGG	M19708	47	14	4.017	2.95E-05	7.35E-03
MHC class II antigen presentation	REACTOME	M705	91	16	3.557	1.87E-04	2.33E-02
Host Interactions of HIV factors	REACTOME	M5283	132	11	3.202	6.81E-04	5.65E-02
Lysosome	KEGG	M11266	121	11	3.111	9.31E-04	5.80E-02
GABA-B receptor activation	REACTOME	M954	38	10	3.008	1.31E-03	6.54E-02
L1CAM interactions	REACTOME	M872	86	17	2.987	1.41E-03	6.54E-02
Vascular smooth muscle contraction	KEGG	M9387	115	22	2.852	2.17E-03	7.73E-02
**Plan 2: Only Males**							
Neurotransmitter receptors and postsynaptic signal transmission	REACTOME	M752	137	25	3.369	3.77E-04	1.02E-01
Transmission across chemical synapses	REACTOME	M15514	186	34	3.287	5.06E-04	1.02E-01
GABA receptor activation	REACTOME	M976	52	11	3.041	1.18E-03	1.06E-01
Phospholipase C-mediated cascade	REACTOME	M856	54	12	2.754	2.94E-03	1.98E-01
**Plan 3: Only Females**							
GABA-B receptor activation	REACTOME	M954	38	11	3.698	1.09E-04	2.66E-02
O-linked glycosylation of mucins	REACTOME	M546	59	10	3.418	3.15E-04	3.86E-02
GABA receptor activation	REACTOME	M976	52	13	3.364	3.84E-04	3.86E-02
extracellular matrix (ECM) regulators	NABA	M3468	238	41	3.361	3.88E-04	3.86E-02
**Plan 5: Non-hypertensive Subjects**							
Retinoblastoma 1 pathway	PID	M279	65	10	3.71	1.04E-04	3.20E-02
Circadian clock	REACTOME	M938	53	12	3.508	2.26E-04	3.48E-02
Alzheimer’s disease	KEGG	M16024	169	24	3.011	1.30E-03	1.34E-01

GSEA: Gene Set Enrichment Analysis; Size: number of genes in the pathway; Count: number of enriched genes in the pathway; KEGG: Kyoto Encyclopedia of Genes and Genomes; REACTOME: REACTOME pathway knowledgebase; PID: Pathway Interaction Database; NABA: Matrisome Project. The false discovery rate thresholds were 0.1, 0.2, 0.05, and 0.15 for plans 1, 2, 3, and 5, respectively.

**Table 5:** Pathway-enrichment of brain-specific methylome-wide association results.

Pathway	Pathway Source	GSEA ID	Size	Count	Z-Score	*p*-Value	*q*-Value
**Plan 1: All Subjects**							
MHC class II antigen presentation	REACTOME	M705	91	14	3.3	4.84E-04	1.07E-01
**Plan 2: Only Males**							
Ubiquitin mediated proteolysis	KEGG	M15247	138	14	3.198	6.91E-04	1.54E-01
Type II diabetes mellitus	KEGG	M19708	47	17	2.89	1.93E-03	2.15E-01
**Plan 3: Only Females**							
MHC class II antigen presentation	REACTOME	M705	91	18	3.138	8.50E-04	1.56E-01
Transport of inorganic cations/anions and amino acids/oligopeptides	REACTOME	M823	94	11	2.849	2.19E-03	2.02E-01
**Plan 4: Hypertensive Subjects**							
DNA repair	REACTOME	M15434	112	10	3.87	5.44E-05	1.26E-02
Type II diabetes mellitus	KEGG	M19708	47	10	3.622	1.46E-04	1.69E-02
Extracellular matrix (ECM) affiliated proteins	NABA	M5880	171	22	3.019	1.27E-03	9.77E-02
**Plan 5: Non-hypertensive Subjects**							
Respiratory electron transport, ATP synthesis by chemiosmotic coupling, and heat production by uncoupling proteins	REACTOME	M1025	98	10	3.851	5.89E-05	1.66E-02
Hematopoietic cell lineage	KEGG	M6856	88	13	3.003	1.33E-03	1.88E-01
The citric acid (TCA) cycle and respiratory electron transport	REACTOME	M516	141	14	2.933	1.68E-03	1.88E-01

GSEA: Gene Set Enrichment Analysis; Size: number of genes in the pathway; Count: number of enriched genes in the pathway; KEGG: Kyoto Encyclopedia of Genes and Genomes; REACTOME: REACTOME pathway knowledgebase; PID: Pathway Interaction Database; NABA: Matrisome Project. The false discovery rate thresholds were 0.2, 0.25, 0.25, 0.1, and 0.2 for plans 1–5, respectively.

## Supplementary Materials

The following are available online at https://www.mdpi.com/2077-0383/9/5/1489/s1, Additional File 1 containing Supporting Acknowledgment, Table S1: Blood-based methylome-wide association results; Table S2: Brain-specific methylome-wide association results; Table S3: Wald chi-square test to compare probes effects between males and females for probes that were significant in blood-based analyses of only one of the two groups; Table S4: Wald chi-square test to compare probes effects between males and females for probes that were significant in brain-specific analyses of only one of the two groups; Table S5: Wald chi-square test to compare probes effects between hypertensive and non-hypertensive subjects for probes that were significant in blood-based analyses of only one of the two groups; Table S6: Wald chi-square test to compare probes effects between hypertensive and non-hypertensive subjects for probes that were significant in brain-specific analyses of only one of the two groups.

## Abbreviations

*ABCA7*	ATP Binding Cassette Subfamily A Member 7
AD	Alzheimer’s Disease
*ADCY8*	Adenylate Cyclase 8
*AIM2*	Absent in Melanoma 2
*ANK1*	Ankyrin 1
*AP2A2*	Adaptor Related Protein Complex 2 Subunit Alpha 2
*APOC1*	Apolipoprotein C1
*APOE*	Apolipoprotein E
*APP*	Amyloid Beta Precursor Protein
Aβ	Amyloid-β
*BIN1*	Bridging Integrator 1
*BPGM*	Bisphosphoglycerate Mutase
*BRD2*	Bromodomain Containing 2
*BUG22*	Basal Body Upregulated Gene 22
*C10orf54*	Chromosome 10 Open Reading Frame 54
*C16orf80*	Chromosome 16 Open Reading Frame 80
*CDH23*	Cadherin Related 23
*CFAP20*	Cilia and Flagella Associated Protein 20
*CHRNA2*	Cholinergic Receptor Nicotinic Alpha 2 Subunit
CHS	Cardiovascular Health Study
*CLIC1*	Chloride Intracellular Channel 1
*CLU*	Clusterin
*CMIP*	C-Maf Inducing Protein
*COL11A2*	Collagen Type XI Alpha 2 Chain
dbGaP	The Database of Genotypes and Phenotypes
*DGUOK*	Deoxyguanosine Kinase
*DUSP22*	Dual Specificity Phosphatase 22
*EBF4*	Early B Cell Factor Family Member 4
*EGFL8*	Epidermal Growth Factor-Like Like Domain Multiple 8
eQTL	Expression Quantitative trait Locus
*FAM193B*	Family with Sequence Similarity 193 Member B
FDR	False Discovery Rate
FHS	Framingham Heart Study
*GABA*	Gamma-Aminobutyric Acid
GRASP	Genome-Wide Repository of Associations Between SNPs and Phenotypes
GSA	Gene Set Analysis
GSA-SNP2	Gene Set Analysis-Single-Nucleotide-Polymorphism-2
GSEA	Gene Set Enrichment Analysis
GWAS	Genome-Wide Association Study
HEIDI	Heterogeneity in Dependent Instruments
*HLA-DPB1*	Human Leukocyte Antigen Class II, DP Beta 1
*HLA-DQA2*	Human Leukocyte Antigen Class II, DQ Alpha 2
*HLA-DQB2*	Human Leukocyte Antigen Class II, DQ Beta 2
*HLA-DRB1*	Human Leukocyte Antigen Class II, DR Beta 1
*HLA-DRB5*	Human Leukocyte Antigen Class II, DR Beta 5
HRS	Health and Retirement Study
HTN	Hypertension
ICD-9	International Classification of Disease codes, Ninth revision
IGR	Inter-Genic Region
*IL-18*	Interleukin 18
*IL-1β*	Interleukin 1 Beta
IRB	Institutional Review Board
*ITIH2*	Inter-Alpha-Trypsin Inhibitor Heavy Chain 2
KEGG	Kyoto Encyclopedia of Genes and Genomes
*KRTAP5-11*	Keratin Associated Protein 5-11
*L1CAM*	L1 Cell Adhesion Molecule
*LECT1*	Leukocyte Cell Derived Chemotaxin 1
LOADFS	Late-Onset Alzheimer's Disease Family Study
*LOC100288866*	Uncharacterized LOC100288866
*LOC154449*	Uncharacterized LOC154449
*MAPT*	Microtubule Associated Protein Tau
MB	Methylene Blue
*MHC*	Major Histocompatibility Complex
mQTL	Methylation Quantitative trait Locus
*MUM*1	Melanoma Ubiquitous Mutated Protein 1
MWA	Methylome-Wide Association
NABA	Matrisome Project
*NANOS2*	Nanos C2HC-Type Zinc Finger 2
*NDUFA4*	NDUFA4 Mitochondrial Complex Associated
*NGFR*	Nerve Growth Factor Receptor
NHGRI-EBI GWAS	National Human Genome Research Institute-European Bioinformatics Institute Genome-Wide Association Studies Catalog
NINCDS-ADRDA	National Institute of Neurological and Communicative Disorders and Stroke of the United States-the Alzheimer’s Disease and Related Disorders Association
*NLRC4*	Nucleotide-Binding Oligomerization Domain, Leucine Rich Repeat and Caspase Recruitment Domain Containing 4
*NLRP3*	Nucleotide-Binding Oligomerization Domain, Leucine Rich Repeat and Pyrin Domain Containing 3
*PHLDA1*	Pleckstrin Homology Like Domain Family A Member 1
PID	Pathway Interaction Database
*PPT2-EGFL8*	Palmitoyl-Protein Thioesterase 2-Epidermal Growth Factor-Like Like Domain Multiple 8 Readthrough
*PSEN1*	Presenilin 1
*PSTK*	Phosphoseryl-TRNA Kinase
*RHBDF2*	Rhomboid 5 Homolog 2
*RPL13*	Ribosomal Protein L13
*SIGLEC12*	Sialic Acid Binding Immunoglobulin Like Lectin 12
*SLC24A4*	Solute Carrier Family 24 Member 4
*SLC25A2*	Solute Carrier Family 25 Member 2
*SLC35C1*	Solute Carrier Family 35 Member C1
*SLC6A7*	Solute Carrier Family 6 Member 7
SMR	Summary Data-Based Mendelian Randomization
SNP	Single-Nucleotide Polymorphism
*SORBS3*	Sorbin And SH3 Domain Containing 3
*SORL1*	Sortilin Related Receptor 1
*ST14*	Suppression of Tumorigenicity 14
TCA	Tricarboxylic Acid
*TOMM40*	Translocase of Outer Mitochondrial Membrane 40
*TREM1*	Triggering Receptor Expressed on Myeloid Cells 1
TWA	Transcriptome-Wide Association
*ZNF394*	Zinc Finger Protein 394
*ZNF598*	Zinc Finger Protein 598

## References

[1] Alzheimer’s Association (2016). 2016 Alzheimer’s disease facts and figures. Alzheimers Dement..

[2] Ridge P.G., Hoyt K.B., Boehme K., Mukherjee S., Crane P.K., Haines J.L., Mayeux R., Farrer L.A., Pericak-Vance M.A., Schellenberg G.D. (2016). Assessment of the genetic variance of late-onset Alzheimer’s disease. Neurobiol. Aging.

[3] Raghavan N., Tosto G. (2017). Genetics of Alzheimer’s disease: The importance of polygenic and epistatic components. Curr. Neurol. Neurosci. Rep..

[4] Leslie R., O’Donnell C.J., Johnson A.D. (2014). GRASP: Analysis of genotype-phenotype results from 1390 genome-wide association studies and corresponding open access database. Bioinformatics.

[5] MacArthur J., Bowler E., Cerezo M., Gil L., Hall P., Hastings E., Junkins H., McMahon A., Milano A., Morales J. (2017). The new NHGRI-EBI Catalog of published genome-wide association studies (GWAS Catalog). Nucleic Acids Res..

[6] Sanchez-Mut J.V., Gräff J. (2015). Epigenetic alterations in Alzheimer’s disease. Front. Behav. Neurosci..

[7] Daviglus M.L., Bell C.C., Berrettini W., Bowen P.E., Connolly E.S., Cox N.J., Dunbar-Jacob J.M., Granieri E.C., Hunt G., McGarry K. (2010). NIH state-of-the-science conference statement: Preventing Alzheimer’s disease and cognitive decline. NIH Consens. State Sci. Statements.

[8] Power M.C., Weuve J., Gagne J.J., McQueen M.B., Viswanathan A., Blacker D. (2011). The association between blood pressure and incident Alzheimer disease: A systematic review and meta-analysis. Epidemiology.

[9] Lahiri D.K., Zawia N.H., Greig N.H., Sambamurti K., Maloney B. (2008). Early-life events may trigger biochemical pathways for Alzheimer’s disease: The “LEARn” model. Biogerontology.

[10] Yokoyama A.S., Rutledge J.C., Medici V. (2017). DNA methylation alterations in Alzheimer’s disease. Environ. Epigenet..

[11] Sanchez-Mut J.V., Aso E., Panayotis N., Lott I., Dierssen M., Rabano A., Urdinguio R.G., Fernandez A.F., Astudillo A., Martin-Subero J.I. (2013). DNA methylation map of mouse and human brain identifies target genes in Alzheimer’s disease. Brain.

[12] Wen K.-X., Miliç J., El-Khodor B., Dhana K., Nano J., Pulido T., Kraja B., Zaciragic A., Bramer W.M., Troup J. (2016). The role of DNA methylation and histone modifications in neurodegenerative diseases: A systematic review. PLoS ONE.

[13] Liu X., Jiao B., Shen L. (2018). The epigenetics of Alzheimer’s disease: Factors and therapeutic implications. Front. Genet..

[14] Iwata A., Nagata K., Hatsuta H., Takuma H., Bundo M., Iwamoto K., Tamaoka A., Murayama S., Saido T., Tsuji S. (2014). Altered CpG methylation in sporadic Alzheimer’s disease is associated with APP and MAPT dysregulation. Hum. Mol. Genet..

[15] Foraker J., Millard S.P., Leong L., Thomson Z., Chen S., Keene C.D., Bekris L.M., Yu C.-E. (2015). The APOE gene is differentially methylated in Alzheimer’s disease. J. Alzheimers Dis..

[16] De Jager P.L., Srivastava G., Lunnon K., Burgess J., Schalkwyk L.C., Yu L., Eaton M.L., Keenan B.T., Ernst J., McCabe C. (2014). Alzheimer’s disease: Early alterations in brain DNA methylation at ANK1, BIN1, RHBDF2 and other loci. Nat. Neurosci..

[17] Lunnon K., Smith R., Hannon E., De Jager P.L., Srivastava G., Volta M., Troakes C., Al-Sarraj S., Burrage J., Macdonald R. (2014). Methylomic profiling implicates cortical deregulation of ANK1 in Alzheimer’s disease. Nat. Neurosci..

[18] Semick S.A., Bharadwaj R.A., Collado-Torres L., Tao R., Shin J.H., Deep-Soboslay A., Weiss J.R., Weinberger D.R., Hyde T.M., Kleinman J.E. (2019). Integrated DNA methylation and gene expression profiling across multiple brain regions implicate novel genes in Alzheimer’s disease. Acta Neuropathol..

[19] Sanchez-Mut J.V., Aso E., Heyn H., Matsuda T., Bock C., Ferrer I., Esteller M. (2014). Promoter hypermethylation of the phosphatase DUSP22 mediates PKA-dependent TAU phosphorylation and CREB activation in Alzheimer’s disease. Hippocampus.

[20] Siegmund K.D., Connor C.M., Campan M., Long T.I., Weisenberger D.J., Biniszkiewicz D., Jaenisch R., Laird P.W., Akbarian S. (2007). DNA methylation in the human cerebral cortex is dynamically regulated throughout the life span and involves differentiated neurons. PLoS ONE.

[21] Lord J., Cruchaga C. (2014). The epigenetic landscape of Alzheimer’s disease. Nat. Neurosci..

[22] Yu L., Chibnik L.B., Srivastava G.P., Pochet N., Yang J., Xu J., Kozubek J., Obholzer N., Leurgans S.E., Schneider J.A. (2015). Association of Brain DNA methylation in SORL1, ABCA7, HLA-DRB5, SLC24A4, and BIN1 with pathological diagnosis of Alzheimer disease. JAMA Neurol..

[23] Fetahu I.S., Ma D., Rabidou K., Argueta C., Smith M., Liu H., Wu F., Shi Y.G. (2019). Epigenetic signatures of methylated DNA cytosine in Alzheimer’s disease. Sci. Adv..

[24] Marioni R.E., Harris S.E., Zhang Q., McRae A.F., Hagenaars S.P., Hill W.D., Davies G., Ritchie C.W., Gale C.R., Starr J.M. (2018). GWAS on family history of Alzheimer’s disease. Transl. Psychiatry.

[25] Zhao T., Hu Y., Zang T., Wang Y. (2019). Integrate GWAS, eQTL, and mQTL data to identify Alzheimer’s disease-related genes. Front. Genet..

[26] Zhu Z., Zhang F., Hu H., Bakshi A., Robinson M.R., Powell J.E., Montgomery G.W., Goddard M.E., Wray N.R., Visscher P.M. (2016). Integration of summary data from GWAS and eQTL studies predicts complex trait gene targets. Nat. Genet..

[27] Nazarian A., Yashin A.I., Kulminski A.M. (2019). Genome-wide analysis of genetic predisposition to Alzheimer’s disease and related sex disparities. Alzheimers Res. Ther..

[28] Nazarian A., Arbeev K.G., Yashkin A.P., Kulminski A.M. (2019). Genetic heterogeneity of Alzheimer’s disease in subjects with and without hypertension. GeroScience.

[29] McRae A.F., Marioni R.E., Shah S., Yang J., Powell J.E., Harris S.E., Gibson J., Henders A.K., Bowdler L., Painter J.N. (2018). Identification of 55,000 Replicated DNA Methylation QTL. Sci. Rep..

[30] Qi T., Wu Y., Zeng J., Zhang F., Xue A., Jiang L., Zhu Z., Kemper K., Yengo L., Zheng Z. (2018). Identifying gene targets for brain-related traits using transcriptomic and methylomic data from blood. Nat. Commun..

[31] Genin E., Hannequin D., Wallon D., Sleegers K., Hiltunen M., Combarros O., Bullido M.J., Engelborghs S., De Deyn P., Berr C. (2011). APOE and Alzheimer disease: A major gene with semi-dominant inheritance. Mol. Psychiatry.

[32] Andersen K., Launer L.J., Dewey M.E., Letenneur L., Ott A., Copeland J.R., Dartigues J.F., Kragh-Sorensen P., Baldereschi M., Brayne C. (1999). Gender differences in the incidence of AD and vascular dementia: The EURODEM Studies. EURODEM incidence research group. Neurology.

[33] Carter C.L., Resnick E.M., Mallampalli M., Kalbarczyk A. (2012). Sex and gender differences in Alzheimer’s disease: Recommendations for future research. J. Womens Health (Larchmt.).

[34] Mayeux R. (2003). Epidemiology of neurodegeneration. Annu. Rev. Neurosci..

[35] Mielke M.M., Vemuri P., Rocca W.A. (2014). Clinical epidemiology of Alzheimer’s disease: Assessing sex and gender differences. Clin. Epidemiol..

[36] Henderson V.W., Buckwalter J.G. (1994). Cognitive deficits of men and women with Alzheimer’s disease. Neurology.

[37] Barnes L.L., Wilson R.S., Bienias J.L., Schneider J.A., Evans D.A., Bennett D.A. (2005). Sex differences in the clinical manifestations of Alzheimer disease pathology. Arch. Gen. Psychiatry.

[38] Faraco G., Iadecola C. (2013). Hypertension: A harbinger of stroke and dementia. Hypertension.

[39] Csiszar A., Tarantini S., Fülöp G.A., Kiss T., Valcarcel-Ares M.N., Galvan V., Ungvari Z., Yabluchanskiy A. (2017). Hypertension impairs neurovascular coupling and promotes microvascular injury: Role in exacerbation of Alzheimer’s disease. GeroScience.

[40] Lloyd-Jones L.R., Holloway A., McRae A., Yang J., Small K., Zhao J., Zeng B., Bakshi A., Metspalu A., Dermitzakis M. (2017). The genetic architecture of gene expression in peripheral blood. Am. J. Hum. Genet..

[41] (2017). GTEx Consortium Genetic effects on gene expression across human tissues. Nature.

[42] Fried L.P., Borhani N.O., Enright P., Furberg C.D., Gardin J.M., Kronmal R.A., Kuller L.H., Manolio T.A., Mittelmark M.B., Newman A. (1991). The cardiovascular health study: Design and rationale. Ann. Epidemiol..

[43] Dawber T.R., Meadors G.F., Moore F.E. (1951). Epidemiological approaches to heart disease: The Framingham study. Am. J. Public Health Nations Health.

[44] Feinleib M., Kannel W.B., Garrison R.J., McNamara P.M., Castelli W.P. (1975). The Framingham offspring study: Design and preliminary data. Prev. Med..

[45] Lee J.H., Cheng R., Graff-Radford N., Foroud T., Mayeux R. (2008). Analyses of the national institute on aging late-onset Alzheimer’s disease family study: Implication of additional loci. Arch. Neurol..

[46] Sonnega A., Faul J.D., Ofstedal M.B., Langa K.M., Phillips J.W., Weir D.R. (2014). Cohort profile: The health and retirement study (HRS). Int. J. Epidemiol..

[47] McKhann G., Drachman D., Folstein M., Katzman R., Price D., Stadlan E.M. (1984). Clinical diagnosis of Alzheimer’s disease: Report of the NINCDS-ADRDA Work Group under the auspices of Department of Health and Human Services Task Force on Alzheimer’s Disease. Neurology.

[48] Purcell S., Neale B., Todd-Brown K., Thomas L., Ferreira M.A.R., Bender D., Maller J., Sklar P., de Bakker P.I.W., Daly M.J. (2007). PLINK: A tool set for whole-genome association and population-based linkage analyses. Am. J. Hum. Genet..

[49] Bates D., Mächler M., Bolker B., Walker S. (2015). Fitting linear mixed-effects models using lme4. J. Stat. Softw..

[50] Mägi R., Morris A.P. (2010). GWAMA: Software for genome-wide association meta-analysis. BMC Bioinform..

[51] Allison P.D. (1999). Comparing logit and probit coefficients across groups. Sociol. Methods Res..

[52] Hannon E., Gorrie-Stone T.J., Smart M.C., Burrage J., Hughes A., Bao Y., Kumari M., Schalkwyk L.C., Mill J. (2018). Leveraging DNA-methylation quantitative-trait loci to characterize the relationship between methylomic variation, gene expression, and complex traits. Am. J. Hum. Genet..

[53] Yoon S., Nguyen H.C.T., Yoo Y.J., Kim J., Baik B., Kim S., Kim J., Kim S., Nam D. (2018). Efficient pathway enrichment and network analysis of GWAS summary data using GSA-SNP2. Nucleic Acids Res..

[54] Subramanian A., Tamayo P., Mootha V.K., Mukherjee S., Ebert B.L., Gillette M.A., Paulovich A., Pomeroy S.L., Golub T.R., Lander E.S. (2005). Gene set enrichment analysis: A knowledge-based approach for interpreting genome-wide expression profiles. Proc. Natl. Acad. Sci. USA.

[55] Kanehisa M., Goto S. (2000). KEGG: Kyoto encyclopedia of genes and genomes. Nucleic Acids Res..

[56] Fabregat A., Jupe S., Matthews L., Sidiropoulos K., Gillespie M., Garapati P., Haw R., Jassal B., Korninger F., May B. (2018). The reactome pathway knowledgebase. Nucleic Acids Res..

[57] Schaefer C.F., Anthony K., Krupa S., Buchoff J., Day M., Hannay T., Buetow K.H. (2009). PID: The pathway interaction database. Nucleic Acids Res..

[58] Naba A., Clauser K.R., Hoersch S., Liu H., Carr S.A., Hynes R.O. (2012). The matrisome: In silico definition and in vivo characterization by proteomics of normal and tumor extracellular matrices. Mol. Cell. Proteom..

[59] Benjamini Y., Hochberg Y. (1995). Controlling the false discovery rate: A practical and powerful approach to multiple testing. J. R. Stat. Soc. Ser. B Methodol..

[60] Smith A.K., Kilaru V., Kocak M., Almli L.M., Mercer K.B., Ressler K.J., Tylavsky F.A., Conneely K.N. (2014). Methylation quantitative trait loci (meQTLs) are consistently detected across ancestry, developmental stage, and tissue type. BMC Genom..

[61] Bollati V., Galimberti D., Pergoli L., Dalla Valle E., Barretta F., Cortini F., Scarpini E., Bertazzi P.A., Baccarelli A. (2011). DNA methylation in repetitive elements and Alzheimer disease. Brain Behav. Immun..

[62] Chang L., Wang Y., Ji H., Dai D., Xu X., Jiang D., Hong Q., Ye H., Zhang X., Zhou X. (2014). Elevation of peripheral BDNF promoter methylation links to the risk of Alzheimer’s disease. PLoS ONE.

[63] Di Francesco A., Arosio B., Falconi A., Micioni Di Bonaventura M.V., Karimi M., Mari D., Casati M., Maccarrone M., D’Addario C. (2015). Global changes in DNA methylation in Alzheimer’s disease peripheral blood mononuclear cells. Brain Behav. Immun..

[64] Nagata T., Kobayashi N., Ishii J., Shinagawa S., Nakayama R., Shibata N., Kuerban B., Ohnuma T., Kondo K., Arai H. (2015). Association between DNA Methylation of the BDNF Promoter Region and Clinical Presentation in Alzheimer’s Disease. Dement. Geriatr. Cogn. Dis. Extra.

[65] Ji H., Wang Y., Liu G., Xu X., Dai D., Chen Z., Zhou D., Zhou X., Han L., Li Y. (2015). OPRK1 promoter hypermethylation increases the risk of Alzheimer’s disease. Neurosci. Lett..

[66] Blass J.P., Hanin I., Barclay L., Kopp U., Reding M.J. (1985). Red blood cell abnormalities in Alzheimer disease. J. Am. Geriatr. Soc..

[67] Sevush S., Jy W., Horstman L.L., Mao W.W., Kolodny L., Ahn Y.S. (1998). Platelet activation in Alzheimer disease. Arch. Neurol..

[68] Etcheberrigaray R., Ibarreta D. (2001). Ionic channels and second messenger alterations in Alzheimer’s disease. Relevance of studies in nonneuronal cells. Rev. Neurol..

[69] Gibson G.E., Huang H.-M. (2002). Oxidative processes in the brain and non-neuronal tissues as biomarkers of Alzheimer’s disease. Front. Biosci..

[70] Catricala S., Torti M., Ricevuti G. (2012). Alzheimer disease and platelets: How’s that relevant. Immun. Ageing.

[71] Kaminsky Y.G., Reddy V.P., Ashraf G.M., Ahmad A., Benberin V.V., Kosenko E.A., Aliev G. (2013). Age-related defects in erythrocyte 2,3-diphosphoglycerate metabolism in dementia. Aging Dis..

[72] Hokama M., Oka S., Leon J., Ninomiya T., Honda H., Sasaki K., Iwaki T., Ohara T., Sasaki T., LaFerla F.M. (2014). Altered expression of diabetes-related genes in Alzheimer’s disease brains: The Hisayama study. Cereb. Cortex.

[73] Hannon E., Weedon M., Bray N., O’Donovan M., Mill J. (2017). Pleiotropic effects of trait-associated genetic variation on DNA methylation: Utility for refining GWAS loci. Am. J. Hum. Genet..

[74] Li H., Wetten S., Li L., St Jean P.L., Upmanyu R., Surh L., Hosford D., Barnes M.R., Briley J.D., Borrie M. (2008). Candidate single-nucleotide polymorphisms from a genomewide association study of Alzheimer disease. Arch. Neurol..

[75] Han M.-R., Schellenberg G.D., Wang L.-S. (2010). Alzheimer’s Disease Neuroimaging Initiative Genome-wide association reveals genetic effects on human Aβ42 and τ protein levels in cerebrospinal fluids: A case control study. BMC Neurol..

[76] Beecham G.W., Hamilton K., Naj A.C., Martin E.R., Huentelman M., Myers A.J., Corneveaux J.J., Hardy J., Vonsattel J.-P., Younkin S.G. (2014). Genome-wide association meta-analysis of neuropathologic features of Alzheimer’s disease and related dementias. PLoS Genet..

[77] Sherva R., Tripodis Y., Bennett D.A., Chibnik L.B., Crane P.K., de Jager P.L., Farrer L.A., Saykin A.J., Shulman J.M., Naj A. (2014). Genome-wide association study of the rate of cognitive decline in Alzheimer’s disease. Alzheimers Dement..

[78] Stelzer G., Rosen N., Plaschkes I., Zimmerman S., Twik M., Fishilevich S., Stein T.I., Nudel R., Lieder I., Mazor Y. (2016). The GeneCards suite: From gene data mining to disease genome sequence analyses. Curr. Protoc. Bioinform..

[79] Keller M.F., Reiner A.P., Okada Y., van Rooij F.J.A., Johnson A.D., Chen M.-H., Smith A.V., Morris A.P., Tanaka T., Ferrucci L. (2014). Trans-ethnic meta-analysis of white blood cell phenotypes. Hum. Mol. Genet..

[80] Ahn H., Kang S.G., Yoon S., Ko H.-J., Kim P.-H., Hong E.-J., An B.-S., Lee E., Lee G.-S. (2017). Methylene blue inhibits NLRP3, NLRC4, AIM2, and non-canonical inflammasome activation. Sci. Rep..

[81] Freeman L.C., Ting J.P.-Y. (2016). The pathogenic role of the inflammasome in neurodegenerative diseases. J. Neurochem..

[82] Liu L., Chan C. (2014). The role of inflammasome in Alzheimer’s disease. Ageing Res. Rev..

[83] Oz M., Lorke D.E., Petroianu G.A. (2009). Methylene blue and Alzheimer’s disease. Biochem. Pharmacol..

[84] Wu P.-J., Liu H.-Y., Huang T.-N., Hsueh Y.-P. (2016). AIM2 inflammasomes regulate neuronal morphology and influence anxiety and memory in mice. Sci. Rep..

[85] Tadiboyina V.T., Rupar A., Atkison P., Feigenbaum A., Kronick J., Wang J., Hegele R.A. (2005). Novel mutation in DGUOK in hepatocerebral mitochondrial DNA depletion syndrome associated with cystathioninuria. Am. J. Med. Genet. A.

[86] Lunnon K., Keohane A., Pidsley R., Newhouse S., Riddoch-Contreras J., Thubron E.B., Devall M., Soininen H., Kłoszewska I., Mecocci P. (2017). Mitochondrial genes are altered in blood early in Alzheimer’s disease. Neurobiol. Aging.

[87] Querfurth H.W., LaFerla F.M. (2010). Alzheimer’s Disease. N. Engl. J. Med..

[88] Braak H., Braak E. (1995). Staging of Alzheimer’s disease-related neurofibrillary changes. Neurobiol. Aging.

[89] Ansoleaga B., Jové M., Schlüter A., Garcia-Esparcia P., Moreno J., Pujol A., Pamplona R., Portero-Otín M., Ferrer I. (2015). Deregulation of purine metabolism in Alzheimer’s disease. Neurobiol. Aging.

[90] Yang W., Tang H., Zhang Y., Tang X., Zhang J., Sun L., Yang J., Cui Y., Zhang L., Hirankarn N. (2013). Meta-analysis followed by replication identifies loci in or near CDKN1B, TET3, CD80, DRAM1, and ARID5B as associated with systemic lupus erythematosus in Asians. Am. J. Hum. Genet..

[91] Wotton C.J., Goldacre M.J. (2017). Associations between specific autoimmune diseases and subsequent dementia: Retrospective record-linkage cohort study, UK. J. Epidemiol. Community Health.

[92] Wirz K.T.S., Bossers K., Stargardt A., Kamphuis W., Swaab D.F., Hol E.M., Verhaagen J. (2013). Cortical beta amyloid protein triggers an immune response, but no synaptic changes in the APPswe/PS1dE9 Alzheimer’s disease mouse model. Neurobiol. Aging.

[93] Yin Z., Raj D., Saiepour N., Van Dam D., Brouwer N., Holtman I.R., Eggen B.J.L., Möller T., Tamm J.A., Abdourahman A. (2017). Immune hyperreactivity of Aβ plaque-associated microglia in Alzheimer’s disease. Neurobiol. Aging.

[94] Mendes Maia T., Gogendeau D., Pennetier C., Janke C., Basto R. (2014). Bug22 influences cilium morphology and the post-translational modification of ciliary microtubules. Biol. Open.

[95] Baird F.J., Bennett C.L. (2013). Microtubule defects & neurodegeneration. J. Genet. Syndr. Gene.

[96] Brunden K.R., Lee V.M.-Y., Smith A.B., Trojanowski J.Q., Ballatore C. (2017). Altered microtubule dynamics in neurodegenerative disease: Therapeutic potential of microtubule-stabilizing drugs. Neurobiol. Dis..

[97] Atamna H., Frey W.H. (2007). Mechanisms of mitochondrial dysfunction and energy deficiency in Alzheimer’s disease. Mitochondrion.

[98] Rodríguez J.J., Verkhratsky A. (2011). Neurogenesis in Alzheimer’s disease. J. Anat..

[99] Hroudová J., Singh N., Fišar Z. (2014). Mitochondrial dysfunctions in neurodegenerative diseases: Relevance to Alzheimer’s disease. Biomed. Res. Int..

[100] Li Y., Sun H., Chen Z., Xu H., Bu G., Zheng H. (2016). Implications of GABAergic Neurotransmission in Alzheimer’s Disease. Front. Aging Neurosci..

[101] Tucsek Z., Noa Valcarcel-Ares M., Tarantini S., Yabluchanskiy A., Fülöp G., Gautam T., Orock A., Csiszar A., Deak F., Ungvari Z. (2017). Hypertension-induced synapse loss and impairment in synaptic plasticity in the mouse hippocampus mimics the aging phenotype: Implications for the pathogenesis of vascular cognitive impairment. GeroScience.

[102] Schetters S.T.T., Gomez-Nicola D., Garcia-Vallejo J.J., Van Kooyk Y. (2018). Neuroinflammation: Microglia and T Cells get ready to tango. Front. Immunol..

[103] Cao W., Zheng H. (2018). Peripheral immune system in aging and Alzheimer’s disease. Mol. Neurodegener..

[104] Chatterjee S., Mudher A. (2018). Alzheimer’s disease and type 2 diabetes: A critical assessment of the shared pathological traits. Front. Neurosci..

