# Supplementary material for: Summary-Based Methylome-Wide Association Analyses Suggest Potential Genetically Driven Epigenetic Heterogeneity of Alzheimer’s Disease
Source: J Clin Med. 2020 May 15;9(5):1489. doi: 10.3390/jcm9051489 (PMC7290473; doi:10.3390/jcm9051489)
Supplement: Supplementary file 1 [file jcm-09-01489-s001.pdf]

Summary-based methylome-wide association analyses suggest potential genetically driven epigenetic heterogeneity of Alzheimer's disease

Alireza Nazarian*, Anatoliy I. Yashin, and Alexander M. Kulminski*

Biodemography of Aging Research Unit, Social Science Research Institute, Duke University, Durham, NC, USA

*Corresponding Authors:

Alireza Nazarian and Alexander M. Kulminski

Duke University

Social Science Research Institute

Biodemography of Aging Research Unit

Erwin Mill Building, 2024 W. Main St.

Durham, NC 27705

Emails: alireza.nazarian@duke.edu and kulminsk@duke.edu

Supporting Acknowledgment
This research was supported by Grants No. P01AG043352, R01AG047310, and R01AG065477 from the National Institute on Aging. The funders had no role in study design, data collection and analysis, decision to publish, or preparation of the manuscript. The content is solely the responsibility of the authors and does not necessarily represent the official views of the National Institutes of Health.
This manuscript was prepared using limited access datasets obtained though dbGaP (accession numbers: phs000287.v5.p1 (CHS), phs000007.v28.p10 (FHS), phs000428.v2.p2 (HRS), and phs000168.v2.p2 (LOADFS) at https://www.ncbi.nlm.nih.gov/gap) and the University of Michigan. Phenotypic HRS data are available publicly and through restricted access from http://hrsonline.isr.umich.edu/index.php?p=data.
The Cardiovascular Health Study (CHS) was supported by contracts HHSN268201200036C, HHSN268200800007C, N01-HC-85079, N01-HC-85080, N01-HC-85081, N01-HC-85082, N01-HC-85083, N01-HC-85084, N01-HC-85085, N01-HC-85086, N01-HC-35129, N01 HC-15103, N01 HC-55222, N01-HC-75150, N01-HC-45133, and N01-HC-85239; grant numbers U01 HL080295 and U01 HL130014 from the National Heart, Lung, and Blood Institute (NHLBI), and R01 AG-023629 from the National Institute on Aging, with additional contribution from the National Institute of Neurological Disorders and Stroke. A full list of principal CHS investigators and institutions can be found at https://chs-nhlbi.org/pi. This manuscript was not prepared in collaboration with CHS investigators and does not necessarily reflect the opinions or views of CHS, or the NHLBI. Support for the genotyping through the CARe Study was provided by NHLBI Contract N01-HC-65226. Additional support for infrastructure was provided by HL105756 and additional genotyping among the African-American cohort was supported in part by HL085251. DNA handling and genotyping at Cedars-Sinai Medical Center was supported in part by National Center for Research Resources grant UL1RR033176, now at the National Center for Advancing Translational Technologies CTSI grant UL1TR000124; in addition to the National Institute of Diabetes and Digestive and Kidney Diseases grant DK063491 to the Southern California Diabetes Endocrinology Research Center.
The Framingham Heart Study (FHS) is conducted and supported by the National Heart, Lung, and Blood Institute (NHLBI) in collaboration with Boston University (Contract No. N01-HC-25195 and HHSN268201500001I). This manuscript was not prepared in collaboration with investigators of the FHS and does not necessarily reflect the opinions or views of the FHS, Boston University, or NHLBI. Funding for SHARe Affymetrix genotyping was provided by NHLBI Contract N02-HL-64278. SHARe Illumina genotyping was provided under an agreement between Illumina and Boston University. Funding for CARe genotyping was provided by NHLBI Contract N01-HC-65226. Funding support for the Framingham Dementia dataset was provided by NIH/NIA grant R01 AG08122. Funding support for the Framingham Inflammatory Markers was provided by NIH grants R01 HL064753, R01 HL076784 and R01 AG028321. Funding support for the Framingham C-reactive Protein dataset was provided by NIH grants R01 HL064753, R01 HL076784 and R01 AG028321. Funding support for the Framingham Adiponectin dataset was provided by NIH/NHLBI grant R01-DK-080739. Funding support for the Framingham Interleukin-6 dataset was provided by NIH grants R01 HL064753, R01 HL076784 and R01 AG028321.
The Health and Retirement Study (HRS) genetic data is sponsored by the Genetics Resource with HRS April 21, 2010, version G Page 5 of 7 National Institute on Aging (grant numbers U01AG009740, RC2AG036495, and RC4AG039029) and was conducted by the University of Michigan. This manuscript

was not prepared in collaboration with HRS investigators and does not necessarily reflect the opinions or views of HRS.
Funding support for the Late Onset Alzheimer's Disease Family Study (LOADFS) was provided through the Division of Neuroscience, NIA. The LOADFS includes a genome-wide association study funded as part of the Division of Neuroscience, NIA. Assistance with phenotype harmonization and genotype cleaning, as well as with general study coordination, was provided by Genetic Consortium for Late Onset Alzheimer's Disease. This manuscript was not prepared in collaboration with LOADFS investigators and does not necessarily reflect the opinions or views of LOADFS.

Tables

Table S1: Blood-based methylome-wide association results

ProbeID	Chr	ProbePos	Gene	SNP	Pos	A1	Freq	P_{GWAS}	P_{mQTL}	b_{SMR}	SE_{SMR}	P_{SMR}	P_{HEIDI}	N_{HEIDI}	Current?	Previous?	Region?
**Plan 1: All Subjects**																	
cg20486877	6p22.1	29626704	*GABBR1*	rs12530421	29581666	C	0.072	2.11E-04	3.47E-66	-0.284	0.057	5.17E-07	5.21E-02	20	N	N	N
cg12893697	11p15.5	970389	*AP2A2*	rs7395773	970503	C	0.481	1.34E-05	0	-0.168	0.031	6.64E-08	4.36E-01	20	S	S	S
cg06064525	11p15.5	970664	*AP2A2*	rs7395773	970503	C	0.481	1.34E-05	0	-0.158	0.029	6.30E-08	5.10E-01	20	S	S	S
cg05626073	11p15.5	971527	*AP2A2*	rs7395773	970503	C	0.481	1.34E-05	1.75E-305	-0.188	0.035	7.49E-08	1.25E-01	20	S	S	S
cg09162537	11p15.5	976950	*AP2A2*	rs7395402	950293	C	0.447	1.54E-05	4.81E-59	-0.352	0.069	3.04E-07	2.03E-01	20	S	S	S
cg21806090	12p13.33	2088868	*CACNA1C*	rs56401832	2074725	C	0.030	2.28E-03	5.18E-165	-0.141	0.028	3.43E-07	3.63E-01	6	N	N	N
cg00222015	12q24.31	121795469	*LINC01089*	rs7964357	121893222	C	0.025	3.21E-04	0	-0.130	0.021	1.54E-09	2.50E-01	20	N	N	N
cg06750524	19q13.32	44906698	*APOE*	rs769449	44906745	A	0.112	2.15E-83	9.38E-15	3.617	0.469	1.24E-14	9.03E-02	10	G	G	G
**Plan 2: Only Males**																	
cg20011360	4p14	37584038	*C4orf19*	rs2276944	37584048	A	0.106	5.94E-04	3.40E-180	0.366	0.058	2.62E-10	3.04E-01	17	N	N	N
cg23395749	5q35.3	177557245	*IGR (FAM193B)*	rs1001530	177558514	G	0.046	4.38E-03	2.55E-26	-0.559	0.109	2.95E-07	2.48E-01	5	N	S	S
cg04627871	6p21.33	31736144	*CLIC1*	rs34585796	31778065	T	0.013	2.46E-02	2.57E-54	0.339	0.059	1.09E-08	1.24E-01	10	N	G	S
cg15598276	6p21.32	32980566	*BRD2*	rs115417321	32973918	C	0.016	6.96E-03	9.03E-38	-0.484	0.073	2.68E-11	2.61E-01	14	N	G	G
cg01991743	6p21.32	33085831	*HLA-DPB1*	rs11551421	33085832	A	0.039	1.59E-03	0	-0.140	0.025	2.79E-08	2.29E-01	20	N	G	G
cg06527438	6q27	170236746	*IGR (LOC154449)*	rs58691983	170238096	A	0.013	2.95E-02	1.44E-25	-0.548	0.098	2.10E-08	4.10E-01	5	N	N	S
cg21205282	6q27	170244045	*IGR (LOC154449)*	rs58691983	170238096	A	0.013	2.95E-02	2.90E-16	-0.693	0.134	2.55E-07	8.05E-02	5	N	N	S
cg22883453	6q27	170244276	*IGR (LOC154449)*	rs58691983	170238096	A	0.013	2.95E-02	1.09E-19	-0.628	0.117	8.38E-08	5.52E-02	4	N	N	S
cg04505845	6q27	170244461	*IGR (LOC154449)*	rs58691983	170238096	A	0.013	2.95E-02	7.99E-22	-0.592	0.108	4.76E-08	2.03E-01	4	N	N	S
cg05526015	6q27	170244757	*IGR (LOC154449)*	rs58691983	170238096	A	0.013	2.95E-02	1.13E-35	-0.463	0.079	4.65E-09	2.18E-01	6	N	N	S
cg24651908	6q27	170245433	*IGR (LOC154449)*	rs58691983	170238096	A	0.013	2.95E-02	1.52E-18	-0.650	0.123	1.18E-07	5.85E-02	4	N	N	S
cg04386759	6q27	170245707	*IGR (LOC154449)*	rs58691983	170238096	A	0.013	2.95E-02	1.36E-25	-0.542	0.097	2.09E-08	5.91E-02	5	N	N	S
cg26180843	6q27	170248014	*IGR (LOC154449)*	rs58691983	170238096	A	0.013	2.95E-02	1.75E-21	-0.584	0.107	5.18E-08	1.14E-01	5	N	N	S
cg12035402	6q27	170250189	*IGR (LOC154449)*	rs58691983	170238096	A	0.013	2.95E-02	1.02E-16	-0.692	0.133	2.16E-07	7.79E-02	5	N	N	S
cg05438220	7q21.13	90643046	*CDK14*	rs17864073	90601202	A	0.022	6.89E-03	0	-0.125	0.023	3.54E-08	2.11E-01	15	N	N	S
cg11399834	8p22	14566901	*SGCZ*	rs7013654	14587192	T	0.047	1.75E-03	6.43E-156	-0.348	0.054	1.20E-10	2.06E-01	20	N	N	G
cg12185975	8q24.22	130761450	*IGR (ADCY8)*	rs79599781	130735526	A	0.057	5.42E-04	2.13E-165	-0.239	0.040	1.73E-09	6.17E-01	20	S	S	S
cg23402755	10p14	7702004	*ITIH2*	rs76129783	7716283	C	0.027	2.98E-03	1.29E-26	-0.453	0.089	3.85E-07	6.42E-01	12	N	G	G
cg14381908	10q11.21	43202369	*RASGEF1A*	rs35718664	43158746	G	0.056	2.03E-03	1.22E-195	0.204	0.040	4.90E-07	2.63E-01	18	N	N	N
cg25422051	11q13.1	65916842	*C11orf68*	rs7940700	65903526	T	0.015	2.87E-02	5.91E-26	-0.658	0.094	2.43E-12	3.54E-01	7	N	N	N
cg15007809	12q21.2	75883289	*IGR (PHLDA1)*	rs11180729	75883290	A	0.014	1.08E-02	1.65E-298	-0.190	0.021	8.73E-20	4.99E-01	10	N	G	G
cg19755435	14q31.3	88006657	*GPR65*	rs1372363	88006834	T	0.076	8.12E-04	2.90E-45	-0.462	0.087	1.04E-07	3.79E-01	20	N	N	S
cg20239174	15q24.1	73780023	*IGR (C15orf59)*	rs1869176	73789242	T	0.010	4.71E-02	3.59E-224	-0.160	0.025	2.42E-10	5.18E-01	7	N	N	N
cg24801087	17q25.3	78485647	*DNAH17,DNAH17-AS1*	rs654154	78485428	C	0.061	3.39E-03	2.53E-45	-0.573	0.106	6.81E-08	2.09E-01	13	N	S	S
cg08778119	17q25.3	78496484	*DNAH17,DNAH17-AS1*	rs12944401	78611646	T	0.028	3.54E-03	5.02E-40	-0.483	0.087	3.23E-08	3.68E-01	4	N	S	S
cg06750524	19q13.32	44906698	*APOE*	rs769449	44906745	A	0.112	7.08E-30	9.38E-15	3.651	0.477	1.92E-14	1.39E-01	10	G	G	G
cg05206559	19q13.32	45913997	*NANOS2*	rs66529687	45914171	A	0.133	1.83E-04	2.84E-41	0.723	0.125	7.67E-09	3.28E-01	20	G	G	G
cg25673584	19q13.32	45914293	*NANOS2*	rs66529687	45914171	A	0.133	1.83E-04	4.40E-30	0.849	0.152	2.45E-08	1.23E-01	20	G	G	G
cg14192299	19q13.32	45914381	*NANOS2*	rs66529687	45914171	A	0.133	1.83E-04	6.71E-42	0.718	0.124	7.30E-09	1.08E-01	20	G	G	G
cg19702802	19q13.32	45914471	*NANOS2*	rs66529687	45914171	A	0.133	1.83E-04	3.22E-39	0.743	0.129	9.10E-09	1.03E-01	20	G	G	G
cg02345758	19q13.43	55837753	*NLRP4,NLRP11*	rs7258704	55849297	T	0.031	5.98E-03	5.63E-73	-0.443	0.067	3.06E-11	5.82E-02	5	N	N	N
**Plan 3: Only Females**																	
cg25325322	2p11.2	84846591	*TRABD2A*	rs116716092	84837385	G	0.034	2.14E-04	1.11E-134	0.244	0.036	1.19E-11	2.20E-01	20	N	N	S
cg18572898	6p21.32	32744326	*HLA-DQA2*	rs7768538	32762044	C	0.426	6.15E-05	0	-0.167	0.032	1.80E-07	1.60E-01	20	S	G	G

cg10218546	6p21.32	32762046	*HLA-DQB2*	rs7768538	32762044	C	0.426	6.15E-05	1.30E-126	-0.304	0.060	3.27E-07	6.43E-02	20	S	G	G
cg02786056	8q13.2	69589075	*SULF1*	rs76431927	69661855	A	0.012	2.19E-02	3.14E-44	-0.421	0.062	1.22E-11	8.98E-01	10	N	N	G
cg00222015	12q24.31	121795469	*LINC01089*	rs7964357	121893222	C	0.025	7.48E-04	0	-0.165	0.025	4.77E-11	1.93E-01	20	N	N	N
cg13328209	12q24.31	124738956	*IGR (SCARB1)*	rs79981325	124732494	T	0.066	9.49E-04	4.32E-34	-0.515	0.102	4.89E-07	9.19E-02	10	N	N	N
cg17632299	13q14.3	52738831	*LECT1*	rs4885947	52735009	C	0.037	2.28E-03	7.51E-54	0.395	0.075	1.67E-07	6.33E-02	20	N	G	G
cg18379295	14q22.1	51859437	*GNG2*	rs17124656	51859367	C	0.217	1.81E-05	7.51E-28	-0.597	0.116	2.79E-07	3.75E-01	20	N	S	G
cg06750524	19q13.32	44906698	*APOE*	rs769449	44906745	A	0.112	6.07E-47	9.38E-15	3.521	0.458	1.58E-14	8.74E-02	10	G	G	G
**Plan 4: Hypertensive Subjects**																	
cg22472863	4q31.22	147096252	*IGR (TTC29)*	rs1507499	147072520	A	0.016	6.68E-04	9.88E-46	-0.314	0.048	7.53E-11	1.78E-01	17	N	N	N
cg23395749	5q35.3	177557245	*IGR (FAM193B)*	rs1001530	177558514	G	0.046	3.36E-04	2.55E-26	-0.484	0.088	3.08E-08	8.77E-02	5	N	S	S
cg06527438	6q27	170236746	*IGR (LOC154449)*	rs58691983	170238096	A	0.013	2.37E-02	1.44E-25	-0.470	0.092	3.40E-07	6.30E-01	5	N	N	S
cg05526015	6q27	170244757	*IGR (LOC154449)*	rs58691983	170238096	A	0.013	2.37E-02	1.13E-35	-0.397	0.075	1.22E-07	1.20E-01	6	N	N	S
cg00222015	12q24.31	121795469	*LINC01089*	rs7964357	121893222	C	0.025	3.54E-03	0	-0.139	0.027	1.58E-07	1.84E-01	20	N	N	N
cg06750524	19q13.32	44906698	*APOE*	rs769449	44906745	A	0.112	4.22E-39	9.38E-15	3.090	0.405	2.23E-14	1.01E-01	10	G	G	G
**Plan 5: Non-hypertensive Subjects**																	
cg15076811	1p36.22	10940981	*IGR (C1orf127)*	rs744921	10938452	A	0.079	3.93E-03	2.26E-43	-0.567	0.112	4.46E-07	7.68E-01	4	N	S	S
cg27535302	1p34.2	42165766	*GUCA2A*	rs116679095	42165766	T	0.013	6.49E-03	3.65E-179	-0.193	0.023	1.38E-16	3.99E-01	13	N	N	G
cg04510459	1p22.1	91582730	*IGR (CDC7)*	rs75735322	91587247	T	0.019	2.71E-03	9.42E-25	0.801	0.115	2.89E-12	3.22E-01	12	N	N	N
cg20306265	1p12	117615002	*FAM46C*	rs2884587	117623474	T	0.170	7.60E-04	3.24E-69	-0.484	0.094	2.81E-07	6.42E-02	20	N	N	N
cg18342183	1q31.1	186763783	*IGR (PLA2G4A)*	rs75463571	186219083	A	0.026	6.25E-03	3.30E-126	-0.402	0.049	2.95E-16	1.40E-01	18	N	S	N
cg23924526	2p21	45771409	*PRKCE*	rs4952770	45771583	A	0.012	4.24E-02	1.41E-27	-0.431	0.078	3.34E-08	9.57E-02	4	N	S	S
cg03063511	2p13.1	73930386	*DGUOK*	rs6737156	73932607	C	0.036	5.62E-03	2.71E-227	-0.247	0.041	2.74E-09	1.09E-01	11	N	N	N
cg25325322	2p11.2	84846591	*TRABD2A*	rs116716092	84837385	G	0.034	1.34E-02	1.11E-134	0.265	0.051	1.86E-07	1.39E-01	20	N	N	S
cg27608154	2q13	111124233	*BCL2L11*	rs3789067	111152506	T	0.061	2.21E-03	4.75E-135	-0.310	0.056	3.03E-08	2.81E-01	11	N	N	G
cg25679431	3p22.3	32147516	*GPD1L*	rs11922351	32130428	G	0.020	3.24E-02	2.00E-182	-0.221	0.043	3.06E-07	1.62E-01	9	N	N	S
cg01317029	3q27.1	184345709	*FAM131A*	rs1049448	184308935	A	0.024	1.91E-02	7.88E-24	0.616	0.122	4.98E-07	1.62E-01	11	N	N	N
cg02951719	4p16.3	1518424	*IGR (NKX1-1)*	rs111388896	1166078	T	0.043	5.15E-03	3.55E-48	-0.490	0.090	5.71E-08	1.46E-01	20	N	N	S
cg05638493	5q31.3	141303800	*SLC25A2*	rs7706206	141322835	A	0.046	2.89E-03	5.84E-34	-0.611	0.105	6.00E-09	2.78E-01	18	N	G	G
cg08631357	5q32	150209647	*SLC6A7*	rs10076748	150209303	A	0.107	1.77E-03	1.54E-193	0.288	0.056	3.18E-07	2.02E-01	20	N	N	G
cg17781669	5q33.1	150488796	*IGR (NDST1)*	rs12519820	150488714	A	0.073	2.10E-03	5.39E-203	0.274	0.050	5.00E-08	1.30E-01	14	N	N	S
cg15538767	5q33.1	150490833	*IGR (NDST1)*	rs12519820	150488714	A	0.073	2.10E-03	1.41E-39	0.552	0.108	3.27E-07	6.20E-01	7	N	N	S
cg19810457	5q35.3	180247356	*MAPK9*	rs77155114	180300706	G	0.016	3.05E-02	4.20E-85	-0.346	0.061	1.16E-08	1.18E-01	6	N	S	S
cg09198601	6p24.3	10318947	*IGR (TFAP2A)*	rs6921983	10318317	T	0.027	1.97E-03	5.34E-22	-0.742	0.117	2.37E-10	3.87E-01	9	N	N	N
cg14466923	6p21.32	33182959	*COL11A2*	rs114068468	33086686	T	0.013	3.09E-02	2.47E-51	-0.226	0.045	4.59E-07	6.83E-02	10	N	G	G
cg17430214	6p21.1	41279166	*TREM1*	rs16894069	40823401	G	0.035	3.43E-03	1.71E-22	0.672	0.123	4.60E-08	7.38E-02	10	N	G	G
cg00531924	6q21	110958687	*GTF3C6*	rs59393044	110958643	C	0.041	6.48E-04	1.54E-16	-1.087	0.188	7.54E-09	1.38E-01	12	N	N	S
cg24039042	6q27	170271859	*IGR (LOC154449)*	rs79920678	170266664	C	0.019	8.48E-03	5.14E-140	0.310	0.044	3.10E-12	1.63E-01	4	N	N	S
cg01766850	6q27	170271878	*IGR (LOC154449)*	rs79920678	170266664	C	0.019	8.48E-03	1.42E-211	0.265	0.038	1.59E-12	2.13E-01	4	N	N	S
cg05973084	6q27	170271951	*IGR (LOC154449)*	rs79920678	170266664	C	0.019	8.48E-03	0	0.193	0.027	6.15E-13	4.25E-01	4	N	N	S
cg09344348	6q27	170271997	*IGR (LOC154449)*	rs79920678	170266664	C	0.019	8.48E-03	0	0.208	0.029	7.58E-13	4.10E-01	4	N	N	S
cg18555277	6q27	170272160	*IGR (LOC154449)*	rs79920678	170266664	C	0.019	8.48E-03	0	0.201	0.028	6.84E-13	3.90E-01	4	N	N	S
cg01209642	6q27	170272267	*IGR (LOC154449)*	rs79920678	170266664	C	0.019	8.48E-03	0	0.200	0.028	6.67E-13	3.49E-01	4	N	N	S
cg21138542	6q27	170272414	*IGR (DLL1)*	rs79920678	170266664	C	0.019	8.48E-03	2.18E-197	0.271	0.038	1.75E-12	1.97E-01	4	N	N	S
cg03899510	6q27	170272602	*IGR (DLL1)*	rs79920678	170266664	C	0.019	8.48E-03	2.06E-45	0.510	0.079	1.07E-10	9.71E-02	4	N	N	S
cg23988375	6q27	170275018	*IGR (DLL1)*	rs79920678	170266664	C	0.019	8.48E-03	1.42E-26	-0.665	0.111	1.97E-09	2.44E-01	4	N	N	S
cg16108844	6q27	170275085	*IGR (DLL1)*	rs79920678	170266664	C	0.019	8.48E-03	1.40E-20	-0.760	0.133	1.06E-08	1.74E-01	3	N	N	S
cg23534345	7p22.1	5639075	*RNF216*	rs852474	5639183	T	0.044	4.34E-04	9.48E-12	1.189	0.234	3.94E-07	5.89E-01	17	N	N	G
cg27103343	7p21.3	10779424	*IGR (NDUFA4)*	rs117926694	10801559	T	0.024	1.18E-02	0	-0.184	0.031	2.96E-09	6.12E-01	20	N	G	G
cg07227187	7q11.21	64192342	*IGR (ZNF735P)*	rs2099421	64206949	G	0.031	6.33E-03	0	-0.149	0.027	3.66E-08	5.84E-01	20	N	S	S

cg01391867	7q22.1	99500248	*ZNF394*	rs117700582	100248819	T	0.026	4.21E-03	2.36E-18	0.787	0.136	7.78E-09	4.44E-01	8	N	G	G
cg23891049	7q33	134679117	*BPGM*	rs73441994	134679118	A	0.021	4.26E-02	1.18E-229	-0.156	0.030	1.70E-07	6.07E-01	4	N	S	S
cg27057461	7q36.3	158343687	*PTPRN2*	rs11975566	158366864	A	0.020	4.60E-03	1.83E-17	-0.750	0.129	6.02E-09	4.46E-01	5	N	N	S
cg04035728	8p21.2	27465950	*CHRNA2*	rs919493	27434040	T	0.139	1.31E-03	1.83E-194	-0.335	0.062	8.00E-08	5.36E-01	20	N	G	G
cg23277389	8q11.21	48921984	*SNAI2*	rs117931199	48936117	T	0.027	1.49E-02	1.33E-26	-0.601	0.118	3.71E-07	3.16E-01	7	N	N	N
cg26687619	8q24.22	133904113	*IGR (ST3GAL1)*	rs55923676	133886617	C	0.020	8.88E-03	5.14E-18	-0.716	0.124	8.59E-09	5.88E-01	3	N	N	S
cg23402755	10p14	7702004	*ITIH2*	rs76129783	7716283	C	0.027	2.75E-04	1.29E-26	-0.726	0.099	2.53E-13	5.25E-01	12	N	G	G
cg23809813	10q23.31	88457915	*RNLS*	rs4933483	88458165	A	0.158	5.70E-04	5.71E-131	0.388	0.071	4.57E-08	5.26E-01	20	N	S	S
cg05399434	10q26.13	121609167	*IGR (FGFR2)*	rs59251821	121607733	T	0.029	7.67E-04	4.58E-26	-0.897	0.119	5.54E-14	5.26E-02	20	N	S	N
cg01628053	10q26.13	121609218	*IGR (FGFR2)*	rs59251821	121607733	T	0.029	7.67E-04	3.77E-23	-0.949	0.131	3.52E-13	1.40E-01	20	N	S	N
cg24635736	10q26.13	122979534	*PSTK*	rs2421140	123027854	A	0.029	8.09E-03	2.67E-77	-0.346	0.060	6.12E-09	7.16E-01	8	N	N	N
cg16255663	10q26.3	129552735	*MGMT*	rs76299173	129527894	T	0.083	1.19E-03	0	-0.204	0.036	8.69E-09	1.05E-01	20	N	S	N
cg17686260	10q26.3	129614500	*MGMT*	rs7893831	129618819	A	0.088	8.70E-04	0	-0.213	0.036	4.05E-09	3.03E-01	20	N	S	N
cg05741225	10q26.3	132103799	*JAKMIP3*	rs78086323	132098502	A	0.022	2.94E-02	3.73E-143	-0.218	0.039	2.91E-08	2.21E-01	6	N	N	N
cg10427719	10q26.3	133334898	*CALY*	rs78775728	133362250	C	0.046	8.30E-03	2.79E-164	-0.262	0.050	1.77E-07	1.21E-01	20	N	N	N
cg02114346	10q26.3	133378038	*PAOX*	rs11101724	133378649	A	0.049	4.14E-03	7.40E-24	0.688	0.137	5.26E-07	1.26E-01	11	N	N	N
cg11435872	11p11.2	45805924	*SLC35C1*	rs2666890	45771472	G	0.066	6.45E-06	1.05E-09	1.519	0.291	1.88E-07	2.73E-01	20	S	N	G
cg05360847	11q13.4	71576873	*IGR (KRTAP5-11)*	rs11827208	71578103	T	0.020	1.70E-03	3.47E-13	-0.942	0.159	3.50E-09	2.02E-01	4	N	N	S
cg02850715	11q24.3	130159317	*ST14*	rs34008994	130165703	T	0.096	1.55E-04	1.21E-26	-0.812	0.138	4.14E-09	7.87E-01	20	N	N	G
cg21029769	11q24.3	130159620	*ST14*	rs34008994	130165703	T	0.096	1.55E-04	4.09E-18	-1.006	0.184	4.58E-08	9.16E-01	20	N	N	G
cg19153300	11q25	134925086	*IGR (LOC283177)*	rs79428444	134915582	T	0.039	5.22E-03	0	-0.201	0.033	1.18E-09	1.89E-01	20	N	N	N
cg21806090	12p13.33	2088868	*CACNA1C*	rs56401832	2074725	C	0.030	2.14E-02	5.18E-165	-0.208	0.040	1.66E-07	5.05E-01	6	N	N	N
cg11915525	12p11.23	27469767	*SMCO2*	rs67786386	27463248	A	0.058	1.30E-03	2.98E-47	-0.502	0.085	3.10E-09	5.37E-01	20	N	N	N
cg21922731	12q13.3	56763346	*HSD17B6*	rs57682836	56774236	T	0.028	1.34E-03	1.86E-14	-0.920	0.167	3.47E-08	8.65E-01	9	N	S	S
cg05979241	12q21.2	77326379	*IGR (E2F7)*	rs10219515	77332811	C	0.026	1.27E-02	8.22E-57	0.424	0.068	3.95E-10	3.58E-01	18	N	N	G
cg24420742	12q21.2	77328028	*IGR (E2F7)*	rs17043304	77308159	G	0.025	1.27E-02	2.67E-80	-0.369	0.057	1.24E-10	7.62E-01	16	N	N	G
cg20090301	12q21.2	77362321	*IGR (E2F7)*	rs10219515	77332811	C	0.026	1.27E-02	1.22E-26	0.624	0.109	9.45E-09	2.98E-01	9	N	N	G
cg20045970	12q24.11	109881214	*GLTP*	rs2287175	110037732	T	0.036	9.24E-03	2.60E-25	-0.704	0.135	1.84E-07	4.11E-01	17	N	N	S
cg17632299	13q14.3	52738831	*LECT1*	rs4885947	52735009	C	0.037	1.23E-03	7.51E-54	0.592	0.085	2.67E-12	1.34E-01	20	N	G	G
cg09557313	13q14.3	52739039	*LECT1*	rs4885947	52735009	C	0.037	1.23E-03	1.46E-40	0.675	0.100	1.37E-11	1.02E-01	20	N	G	G
cg13822911	13q33.2	106376925	*LINC00460*	rs1351059	106378937	A	0.163	4.74E-04	3.61E-38	-0.649	0.125	2.03E-07	5.71E-02	16	N	S	S
cg05099145	16p13.3	1746332	*MAPK8IP3*	rs114127401	1746293	A	0.011	9.72E-03	6.95E-17	-1.613	0.196	2.07E-16	8.07E-01	8	N	S	G
cg11220950	16p13.3	1992692	*SYNGR3*	rs11542302	1986934	C	0.181	7.26E-05	8.47E-17	-1.153	0.223	2.27E-07	5.53E-02	17	N	S	G
cg04519296	16p13.3	1993014	*SYNGR3*	rs11248905	1999727	T	0.181	4.88E-05	8.21E-26	-1.037	0.176	3.88E-09	9.73E-02	17	N	S	G
cg09397293	16p13.3	2005032	*ZNF598*	rs72766639	2005819	A	0.174	1.69E-04	5.78E-51	0.688	0.116	3.06E-09	2.85E-01	20	N	S	G
cg26804891	16p13.3	2005241	*ZNF598*	rs11248905	1999727	T	0.181	4.88E-05	3.56E-98	0.539	0.080	1.62E-11	7.60E-02	20	N	S	G
cg08576185	16p13.3	2005683	*ZNF598*	rs72766639	2005819	A	0.174	1.69E-04	4.06E-44	0.740	0.126	4.76E-09	3.59E-01	20	N	S	G
cg10470208	16p13.3	2008700	*ZNF598*	rs1058474	1998795	T	0.181	6.82E-05	6.56E-19	1.112	0.209	1.02E-07	7.58E-02	14	N	S	G
cg06998361	16q21	58110599	*IGR (C16orf80)*	rs10445026	58109349	G	0.069	5.00E-04	5.61E-97	-0.442	0.069	1.35E-10	2.53E-01	20	N	S	S
cg15638306	17q21.33	49506292	*NGFR,LOC100288866*	rs55759007	49506219	G	0.109	5.49E-04	7.80E-19	-1.047	0.206	3.96E-07	3.09E-01	15	N	G	G
cg25674938	19p13.3	1360982	*MUM1*	rs3760991	1402998	A	0.026	2.54E-02	1.78E-101	-0.315	0.053	2.97E-09	2.54E-01	8	N	G	G
cg06750524	19q13.32	44906698	*APOE*	rs769449	44906745	A	0.112	8.19E-30	9.38E-15	4.407	0.573	1.39E-14	6.84E-02	10	G	G	G
cg04698472	19q13.41	51497571	*SIGLEC12*	rs56102305	51498028	A	0.035	3.19E-03	0	-0.170	0.027	1.62E-10	8.24E-02	20	N	G	G
cg18795732	19q13.42	55549968	*IGR (SBK3)*	rs61747393	55500206	T	0.027	1.08E-02	2.08E-84	-0.310	0.051	1.12E-09	3.97E-01	8	N	N	S
cg02345758	19q13.43	55837753	*NLRP4,NLRP11*	rs7258704	55849297	T	0.031	9.01E-03	5.63E-73	-0.463	0.070	2.75E-11	7.09E-01	5	N	N	N
cg23062096	20p13	2747930	*EBF4*	rs16990611	2747924	G	0.121	2.50E-04	5.77E-20	0.912	0.180	3.89E-07	5.32E-01	6	N	G	G
cg18120111	21q22.3	42496937	*RSPH1*	rs73227543	42503966	G	0.192	7.00E-04	2.03E-108	-0.400	0.078	2.64E-07	2.44E-01	20	N	S	G
cg20460554	21q22.3	42497150	*RSPH1*	rs73227543	42503966	G	0.192	7.00E-04	0	-0.273	0.052	1.57E-07	1.21E-01	20	N	S	G
cg04903116	22q11.21	21443870	*HIC2*	rs78037651	21646689	G	0.023	8.82E-03	8.21E-82	-0.356	0.058	9.59E-10	3.16E-01	13	N	N	S

cg22846809	22q11.21	21646831	*IGR (SDF2L1)*	rs78037651	21646689	G	0.023	8.82E-03	4.16E-91	0.340	0.055	7.80E-10	3.66E-01	13	N	N	S

Genomic coordinates are based on Human Genome version 38 (hg38).

Chr: chromosomal region (i.e., cytogenetic band); ProbePos: probe position; Gene: the gene or closest gene corresponding to the probe; IGR: intergenic region; SNP: top methylation quantitative trait locus (mQTL); Pos: SNP position; A1/Freq: SNP's effect allele and its frequency; P_{GWAS}: p-value of the SNP in genome-wide association meta-analysis; P_{mQTL}: p-value of the SNP in mQTLs analysis; b_{SMR}, SE_{SMR}, and P_{SMR}: beta coefficient, its standard error, and p-value of the probe in summary data-based Mendelian randomization (SMR) test; P_{HEIDI}: p-value of the heterogeneity in dependent instruments (HEIDI) test; N_{HEIDI}: number of single-nucleotide polymorphisms used for HEIDI test; Current?: whether there is any AD-associated SNP within ±1 Mb of the probe in the current genome-wide meta-analysis (N: None, G: SNP with P_{GWAS} < 5E-08, and S: SNP with 5E-08 ≤ P_{GWAS} < 5E-06); Previous?: whether there is any AD-associated SNP within ±1 Mb of the probe in previous GWAS (N: None, G: SNP with P_{GWAS} < 5E-08, and S: SNP with 5E-08 ≤ P_{GWAS} < 5E-06); Region?: whether there is any AD-associated SNP within the chromosomal region (i.e., cytogenetic band) corresponding to the probe (N: None, G: SNP with P_{GWAS} < 5E-08, and S: SNP with 5E-08 ≤ P_{GWAS} < 5E-06).

Table S2: Brain-specific methylome-wide association results

ProbeID	Chr	ProbePos	Gene	SNP	Pos	A1	Freq	P_{GWAS}	P_{mQTL}	b_{SMR}	SE_{SMR}	P_{SMR}	P_{HEIDI}	N_{HEIDI}	Current?	Previous?	Region?
**Plan 1: All Subjects**																	
cg23395749	5q35.3	177557245	*IGR (FAM193B)*	rs1001530	177558514	A	0.954	4.88E-05	2.34E-15	-0.726	0.139	1.90E-07	7.31E-02	5	N	S	S
cg02613937	19q13.32	44892040	*TOMM40*	rs157582	44892962	C	0.789	3.73E-63	1.84E-08	-3.518	0.629	2.28E-08	7.24E-01	7	G	G	G
**Plan 2: Only Males**																	
cg17249452	2q37.3	239609813	*IGR (LOC150935)*	rs72998459	239625495	C	0.933	5.39E-04	1.00E-300	-0.192	0.031	9.13E-10	8.18E-01	13	N	S	S
cg23902610	2q37.3	239609910	*IGR (LOC150935)*	rs72998459	239625495	C	0.933	5.39E-04	3.45E-31	-0.355	0.065	4.35E-08	1.85E-01	15	N	S	S
cg23379309	9q22.33	99295046	*IGR (NAMA)*	rs431892	99293775	C	0.985	2.27E-02	2.67E-34	-0.755	0.126	2.18E-09	7.02E-02	5	N	N	S
cg03080043	16q24.2	88484856	*ZFPM1*	rs34353181	88481573	A	0.050	2.49E-03	7.88E-19	-0.567	0.106	9.96E-08	8.10E-01	4	N	S	S
cg02613937	19q13.32	44892040	*TOMM40*	rs157582	44892962	C	0.789	9.84E-24	1.84E-08	-3.636	0.656	3.06E-08	7.90E-01	7	G	G	G
cg05206559	19q13.32	45913997	*NANOS2*	rs66529687	45914171	G	0.867	1.83E-04	5.86E-298	0.272	0.043	2.96E-10	8.50E-01	19	G	G	G
**Plan 3: Only Females**																	
cg12833011	5q35.1	170503531	*KCNIP1*	rs57212455	170519236	T	0.905	5.78E-04	1.86E-266	0.200	0.038	2.10E-07	8.90E-02	5	N	N	N
cg04322111	6p21.32	32761987	*HLA-DQB2*	rs7768538	32762044	A	0.574	6.15E-05	0	-0.201	0.039	2.21E-07	8.61E-02	20	S	G	G
cg10218546	6p21.32	32762046	*HLA-DQB2*	rs7768538	32762044	A	0.574	6.15E-05	0	-0.198	0.038	2.18E-07	8.32E-02	20	S	G	G
cg02613937	19q13.32	44892040	*TOMM40*	rs157582	44892962	C	0.789	2.17E-35	1.84E-08	-3.371	0.607	2.77E-08	7.08E-01	7	G	G	G
**Plan 4: Hypertensive Subjects**																	
cg11750962	1p36.22	11134621	*MTOR*	rs2275528	11139092	T	0.979	3.00E-03	2.11E-41	-0.411	0.075	5.14E-08	9.76E-02	4	N	S	S
cg12833011	5q35.1	170503531	*KCNIP1*	rs57212455	170519236	T	0.905	2.77E-04	1.86E-266	0.215	0.038	1.91E-08	4.09E-01	5	N	N	N
cg23395749	5q35.3	177557245	*IGR (FAM193B)*	rs1001530	177558514	A	0.954	3.36E-04	2.34E-15	-0.791	0.157	5.17E-07	1.01E-01	5	N	S	S
cg02613937	19q13.32	44892040	*TOMM40*	rs157582	44892962	C	0.789	8.91E-29	1.84E-08	-2.933	0.532	3.51E-08	6.40E-01	7	G	G	G
**Plan 5: Non-hypertensive Subjects**																	
cg09127005	1p34.1	43950718	*IPO13*	rs3791118	43963230	G	0.962	8.98E-04	3.11E-13	-0.668	0.123	5.13E-08	5.72E-01	11	N	S	N
cg11003133	1q23.1	159076601	*AIM2*	rs16841642	159077008	G	0.952	5.30E-03	6.30E-82	-0.312	0.062	4.62E-07	3.40E-01	18	N	S	N
cg04198912	1q32.1	205567693	*MFSD4*	rs6674683	205532045	C	0.971	1.41E-03	3.99E-10	0.654	0.123	1.08E-07	7.41E-01	3	N	N	S
cg03706720	3q27.1	184513150	*IGR (EPHB3)*	rs28729155	184513380	A	0.174	5.48E-05	2.20E-291	-0.328	0.048	1.05E-11	2.27E-01	20	N	N	N
cg06691616	4q31.1	138609944	*IGR (LINC00499)*	rs17317347	138607917	C	0.960	1.59E-03	2.20E-17	0.522	0.094	2.84E-08	1.65E-01	8	N	S	G
cg08631357	5q32	150209647	*SLC6A7*	rs10076748	150209303	C	0.893	1.77E-03	2.82E-295	0.230	0.045	2.76E-07	2.24E-01	18	N	N	G
cg02769614	6p21.32	32167275	*PPT2-EGFL8,EGFL8*	rs17493811	32177622	C	0.970	6.41E-04	5.16E-18	0.535	0.089	1.58E-09	2.46E-01	3	N	G	G
cg08288383	7q21.3	98051318	*IGR (LMTK2)*	rs12538837	98055156	C	0.266	1.86E-04	4.49E-27	0.658	0.128	2.86E-07	8.87E-02	19	N	S	S
cg10308629	7q33	134670051	*BPGM*	rs73439998	134663724	C	0.979	3.01E-02	9.28E-48	-0.520	0.101	2.88E-07	2.57E-01	3	N	S	S
cg04388919	7q36.1	149422042	*IGR (ZNF777)*	rs10248356	149423091	G	0.837	2.01E-02	4.78E-15	1.421	0.229	4.97E-10	2.44E-01	10	N	S	S
cg07189157	7q36.1	149422316	*IGR (ZNF777)*	rs10248356	149423091	G	0.837	2.01E-02	1.30E-22	1.172	0.166	1.52E-12	4.69E-01	9	N	S	S
cg24635736	10q26.13	122979534	*PSTK*	rs13328826	122992107	A	0.970	6.26E-03	2.48E-20	-0.374	0.072	1.68E-07	8.24E-01	3	N	N	N
cg25214093	10q26.13	124447241	*IGR (NKX1-2)*	rs1399928	124446693	T	0.147	7.38E-04	1.89E-296	0.260	0.047	3.31E-08	5.00E-01	10	N	N	N
cg15567360	11q13.4	71611653	*IGR (KRTAP5-11)*	rs11827208	71578103	C	0.980	1.70E-03	9.66E-10	-0.679	0.130	1.67E-07	3.71E-01	3	N	N	S
cg03249590	12q13.3	56646415	*ATP5B,SNORD59A*	rs12321649	56621349	T	0.985	3.22E-02	4.91E-28	0.331	0.059	2.00E-08	5.50E-02	3	N	S	S
cg06679265	12q13.3	56646420	*ATP5B,SNORD59A*	rs12321649	56621349	T	0.985	3.22E-02	2.16E-23	0.359	0.066	4.72E-08	2.41E-01	3	N	S	S
cg09557313	13q14.3	52739039	*LECT1*	rs4885961	52755200	C	0.960	4.63E-03	6.93E-31	0.547	0.103	1.06E-07	5.67E-01	7	N	G	G
cg04870949	13q34	113455542	*IGR (DCUN1D2)*	rs9577553	113455830	C	0.870	1.83E-03	3.84E-282	-0.270	0.053	4.21E-07	1.29E-01	18	N	S	S
cg07011318	16p13.3	2004943	*ZNF598*	rs72766639	2005819	G	0.826	1.69E-04	0	0.291	0.046	1.96E-10	1.12E-01	17	N	S	G
cg09397293	16p13.3	2005032	*ZNF598*	rs72766639	2005819	G	0.826	1.69E-04	0	0.282	0.044	1.86E-10	1.13E-01	18	N	S	G
cg05211189	16p13.3	2005402	*ZNF598*	rs11542302	1986934	T	0.819	7.26E-05	0	0.283	0.043	7.47E-11	1.01E-01	18	N	S	G
cg08576185	16p13.3	2005683	*ZNF598*	rs72766639	2005819	G	0.826	1.69E-04	0	0.295	0.046	2.00E-10	9.02E-02	16	N	S	G
cg06998361	16q21	58110599	*IGR (C16orf80)*	rs74019790	58107923	T	0.931	5.00E-04	4.77E-20	-0.591	0.109	5.49E-08	6.81E-01	11	N	S	S
cg10191399	16q23.3	81693451	*CMIP*	rs72831117	81693634	G	0.914	1.04E-03	0	0.246	0.041	1.54E-09	8.86E-02	20	N	G	G

cg02613937	19q13.32	44892040	*TOMM40*	rs157582	44892962	C	0.789	2.71E-24	1.84E-08	-4.421	0.793	2.50E-08	6.07E-01	7	G	G	G
cg20002045	21q22.3	42499561	*SLC37A1*	rs73227543	42503966	G	0.192	7.00E-04	5.50E-63	-0.456	0.090	4.49E-07	1.87E-01	20	N	S	G
cg01716252	21q22.3	45132690	*ADARB1*	rs439884	45114025	C	0.903	8.78E-04	3.49E-19	0.827	0.164	4.46E-07	1.32E-01	17	N	S	G

Please see the description provided below Table S1.

Table S3: Wald chi-square test to compare probes effects between males and females for probes that were significant in blood-based analyses of only one of the two groups

Significant Probes				Males					Females					Comparison		
**ProbeID**	**Chr**	**ProbePos**	**Gene**	**b_{SMR}**	**SE_{SMR}**	**P_{SMR}**	**P_{HEIDI}**	**N_{HEIDI}**	**b_{SMR}**	**SE_{SMR}**	**P_{SMR}**	**P_{HEIDI}**	**N_{HEIDI}**	**Chisq**	**P**	**Sig?**
**Plan 2: Only Males**																
cg20011360	4p14	37584038	*C4orf19*	0.366	0.058	2.62E-10	3.04E-01	17	0.075	0.059	2.03E-01	1.72E-02	17	12.518	4.03E-04	Y
cg23395749	5q35.3	177557245	*IGR (FAM193B)*	-0.559	0.109	2.95E-07	2.48E-01	5	-0.396	0.087	4.89E-06	3.05E-02	5	1.385	2.39E-01	N
cg04627871	6p21.33	31736144	*CLIC1*	0.339	0.059	1.09E-08	1.24E-01	10	-0.157	0.200	4.32E-01	1.44E-01	10	5.670	1.73E-02	N
cg15598276	6p21.32	32980566	*BRD2*	-0.484	0.073	2.68E-11	2.61E-01	14	-0.078	0.115	4.97E-01	7.35E-01	14	8.931	2.80E-03	N
cg01991743	6p21.32	33085831	*HLA-DPB1*	-0.140	0.025	2.79E-08	2.29E-01	20	-0.017	0.033	6.04E-01	6.31E-01	20	8.742	3.11E-03	N
cg06527438	6q27	170236746	*IGR (LOC154449)*	-0.548	0.098	2.10E-08	4.10E-01	5	-0.188	0.117	1.08E-01	3.73E-01	5	5.579	1.82E-02	N
cg21205282	6q27	170244045	*IGR (LOC154449)*	-0.693	0.134	2.55E-07	8.05E-02	5	-0.237	0.149	1.11E-01	1.68E-01	5	5.148	2.33E-02	N
cg22883453	6q27	170244276	*IGR (LOC154449)*	-0.628	0.117	8.38E-08	5.52E-02	4	-0.215	0.135	1.10E-01	1.81E-01	4	5.349	2.07E-02	N
cg04505845	6q27	170244461	*IGR (LOC154449)*	-0.592	0.108	4.76E-08	2.03E-01	4	-0.203	0.127	1.09E-01	2.38E-01	4	5.446	1.96E-02	N
cg05526015	6q27	170244757	*IGR (LOC154449)*	-0.463	0.079	4.65E-09	2.18E-01	6	-0.159	0.098	1.07E-01	2.65E-01	6	5.807	1.60E-02	N
cg24651908	6q27	170245433	*IGR (LOC154449)*	-0.650	0.123	1.18E-07	5.85E-02	4	-0.223	0.140	1.10E-01	1.32E-01	4	5.290	2.15E-02	N
cg04386759	6q27	170245707	*IGR (LOC154449)*	-0.542	0.097	2.09E-08	5.91E-02	5	-0.186	0.116	1.08E-01	1.70E-01	5	5.580	1.82E-02	N
cg26180843	6q27	170248014	*IGR (LOC154449)*	-0.584	0.107	5.18E-08	1.14E-01	5	-0.200	0.125	1.09E-01	1.90E-01	5	5.432	1.98E-02	N
cg12035402	6q27	170250189	*IGR (LOC154449)*	-0.692	0.133	2.16E-07	7.79E-02	5	-0.237	0.149	1.11E-01	2.37E-01	5	5.179	2.29E-02	N
cg05438220	7q21.13	90643046	*CDK14*	-0.125	0.023	3.54E-08	2.11E-01	15	0.026	0.037	4.85E-01	3.21E-01	15	12.045	5.19E-04	Y
cg11399834	8p22	14566901	*SGCZ*	-0.348	0.054	1.20E-10	2.06E-01	20	-0.004	0.062	9.44E-01	5.44E-01	20	17.481	2.90E-05	Y
cg12185975	8q24.22	130761450	*IGR (ADCY8)*	-0.239	0.040	1.73E-09	6.17E-01	20	0.067	0.058	2.45E-01	1.62E-01	20	18.999	1.31E-05	Y
cg23402755	10p14	7702004	*ITIH2*	-0.453	0.089	3.85E-07	6.42E-01	12	-0.066	0.113	5.55E-01	4.38E-01	12	7.218	7.22E-03	N
cg14381908	10q11.21	43202369	*RASGEF1A*	0.204	0.040	4.90E-07	2.63E-01	18	0.095	0.045	3.62E-02	5.45E-01	18	3.181	7.45E-02	N
cg25422051	11q13.1	65916842	*C11orf68*	-0.658	0.094	2.43E-12	3.54E-01	7	0.115	0.161	4.75E-01	7.53E-02	7	17.242	3.29E-05	Y
cg15007809	12q21.2	75883289	*IGR (PHLDA1)*	-0.190	0.021	8.73E-20	4.99E-01	10	-0.033	0.039	4.02E-01	2.46E-01	10	12.674	3.71E-04	Y
cg19755435	14q31.3	88006657	*GPR65*	-0.462	0.087	1.04E-07	3.79E-01	20	0.002	0.093	9.84E-01	9.65E-01	20	13.282	2.68E-04	Y
cg20239174	15q24.1	73780023	*IGR (C15orf59)*	-0.160	0.025	2.42E-10	5.18E-01	7	0.018	0.057	7.51E-01	5.17E-02	7	8.047	4.56E-03	N
cg24801087	17q25.3	78485647	*DNAH17,DNAH17-AS1*	-0.573	0.106	6.81E-08	2.09E-01	13	0.197	0.152	1.94E-01	2.39E-01	13	17.293	3.20E-05	Y
cg08778119	17q25.3	78496484	*DNAH17,DNAH17-AS1*	-0.483	0.087	3.23E-08	3.68E-01	4	0.001	0.111	9.90E-01	1.58E-01	4	11.712	6.21E-04	Y
cg05206559	19q13.32	45913997	*NANOS2*	0.723	0.125	7.67E-09	3.28E-01	20	-0.048	0.138	7.26E-01	1.16E-01	20	17.132	3.49E-05	Y
cg25673584	19q13.32	45914293	*NANOS2*	0.849	0.152	2.45E-08	1.23E-01	20	-0.057	0.162	7.26E-01	2.33E-01	20	16.586	4.65E-05	Y
cg14192299	19q13.32	45914381	*NANOS2*	0.718	0.124	7.30E-09	1.08E-01	20	-0.048	0.137	7.26E-01	2.28E-01	20	17.155	3.45E-05	Y
cg19702802	19q13.32	45914471	*NANOS2*	0.743	0.129	9.10E-09	1.03E-01	20	-0.050	0.142	7.26E-01	1.95E-01	20	17.054	3.63E-05	Y
cg02345758	19q13.43	55837753	*NLRP4,NLRP11*	-0.443	0.067	3.06E-11	5.82E-02	5	0.001	0.096	9.90E-01	7.59E-01	5	14.402	1.48E-04	Y
**Plan 3: Only Females**																
cg25325322	2p11.2	84846591	*TRABD2A*	0.055	0.063	3.85E-01	4.69E-01	20	0.244	0.036	1.19E-11	2.20E-01	20	6.793	9.15E-03	N
cg18572898	6p21.32	32744326	*HLA-DQA2*	-0.014	0.046	7.65E-01	5.29E-01	20	-0.167	0.032	1.80E-07	1.60E-01	20	7.381	6.59E-03	N
cg10218546	6p21.32	32762046	*HLA-DQB2*	-0.025	0.084	7.65E-01	5.84E-01	20	-0.304	0.060	3.27E-07	6.43E-02	20	7.277	6.99E-03	N
cg02786056	8q13.2	69589075	*SULF1*	0.091	0.228	6.91E-01	1.02E-02	10	-0.421	0.062	1.22E-11	8.98E-01	10	4.685	3.04E-02	N
cg00222015	12q24.31	121795469	*LINC01089*	-0.106	0.034	1.51E-03	1.83E-01	20	-0.165	0.025	4.77E-11	1.93E-01	20	1.961	1.61E-01	N
cg13328209	12q24.31	124738956	*IGR (SCARB1)*	0.201	0.196	3.05E-01	8.15E-01	10	-0.515	0.102	4.89E-07	9.19E-02	10	10.453	1.22E-03	Y
cg17632299	13q14.3	52738831	*LECT1*	0.128	0.140	3.62E-01	1.81E-02	20	0.395	0.075	1.67E-07	6.33E-02	20	2.805	9.40E-02	N
cg18379295	14q22.1	51859437	*GNG2*	0.448	0.217	3.89E-02	4.01E-01	20	-0.597	0.116	2.79E-07	3.75E-01	20	18.038	2.17E-05	Y

Genomic coordinates are based on Human Genome version 38 (hg38).

Chr: chromosomal region (i.e., cytogenetic band); ProbePos: probe position; Gene: the gene or closest gene corresponding to the probe; b_{SMR}, SE_{SMR}, and P_{SMR}: beta coefficient, its standard error, and p-value of the probe in summary data-based Mendelian randomization (SMR) test; P_{HEIDI}: p-value of the heterogeneity in dependent instruments (HEIDI) test; N_{HEIDI}: number of single-nucleotide polymorphisms used for HEIDI test; Chisq and P: chi-square measure and its p-value from comparing the b_{SMR} of probe between the two groups; Sig?: if the probe has group-specific effects (Y: YES, N: NO) at the Bonferroni-adjusted significance level (i.e., 0.05/n.probes, where n.probes is the total number of probes in the Table).

Table S4: Wald chi-square test to compare probes effects between males and females for probes that were significant in brain-specific analyses of only one of the two groups

Significant Probes				Males					Females					Comparison		
ProbeID	Chr	ProbePos	Gene	b_{SMR}	SE_{SMR}	P_{SMR}	P_{HEIDI}	N_{HEIDI}	b_{SMR}	SE_{SMR}	P_{SMR}	P_{HEIDI}	N_{HEIDI}	Chisq	P	Sig?
**Plan 2: Only Males**																
cg17249452	2q37.3	239609813	*IGR (LOC150935)*	-0.192	0.031	9.13E-10	8.18E-01	13	0.082	0.055	1.36E-01	7.01E-01	13	18.724	1.51E-05	Y
cg23902610	2q37.3	239609910	*IGR (LOC150935)*	-0.355	0.065	4.35E-08	1.85E-01	15	0.152	0.102	1.39E-01	4.56E-01	15	17.456	2.94E-05	Y
cg23379309	9q22.33	99295046	*IGR (NAMA)*	-0.755	0.126	2.18E-09	7.02E-02	5	0.423	0.247	8.71E-02	6.56E-02	5	18.010	2.20E-05	Y
cg03080043	16q24.2	88484856	*ZFPM1*	-0.567	0.106	9.96E-08	8.10E-01	4	-0.042	0.114	7.08E-01	9.35E-01	4	11.359	7.51E-04	Y
cg05206559	19q13.32	45913997	*NANOS2*	0.272	0.043	2.96E-10	8.50E-01	19	-0.018	0.052	7.26E-01	1.81E-01	19	18.469	1.73E-05	Y
**Plan 3: Only Females**																
cg12833011	5q35.1	170503531	*KCNIP1*	-0.017	0.063	7.91E-01	1.70E-01	5	0.200	0.038	2.10E-07	8.90E-02	5	8.514	3.53E-03	Y
cg04322111	6p21.32	32761987	*HLA-DQB2*	-0.017	0.056	7.65E-01	5.04E-01	20	-0.201	0.039	2.21E-07	8.61E-02	20	7.346	6.72E-03	N
cg10218546	6p21.32	32762046	*HLA-DQB2*	-0.016	0.055	7.65E-01	4.98E-01	20	-0.198	0.038	2.18E-07	8.32E-02	20	7.349	6.71E-03	N

Please see the description provided below Table S3.

Table S5: Wald chi-square test to compare probes effects between hypertensive and non-hypertensive subjects for probes that were significant in blood-based analyses of only one of the two groups

Significant Probes				Hypertensive Subjects					Non-hypertensive Subjects					Comparison		
ProbeID	Chr	ProbePos	Gene	b_{SMR}	SE_{SMR}	P_{SMR}	P_{HEIDI}	N_{HEIDI}	b_{SMR}	SE_{SMR}	P_{SMR}	P_{HEIDI}	N_{HEIDI}	Chisq	P	Sig?
**Plan 4: Hypertensive Subjects**																
cg22472863	4q31.22	147096252	*IGR (TTC29)*	-0.314	0.048	7.53E-11	1.78E-01	17	-0.032	0.116	7.84E-01	1.56E-02	17	5.071	2.43E-02	N
cg23395749	5q35.3	177557245	*IGR (FAM193B)*	-0.484	0.088	3.08E-08	8.77E-02	5	-0.520	0.113	4.38E-06	5.79E-04	5	0.060	8.06E-01	N
cg06527438	6q27	170236746	*IGR (LOC154449)*	-0.470	0.092	3.40E-07	6.30E-01	5	-0.141	0.148	3.40E-01	1.29E-02	5	3.543	5.98E-02	N
cg05526015	6q27	170244757	*IGR (LOC154449)*	-0.397	0.075	1.22E-07	1.20E-01	6	-0.119	0.125	3.40E-01	3.27E-02	6	3.620	5.71E-02	N
cg00222015	12q24.31	121795469	*LINC01089*	-0.139	0.027	1.58E-07	1.84E-01	20	-0.104	0.040	1.03E-02	5.26E-01	20	0.547	4.60E-01	N
**Plan 5: Non-hypertensive Subjects**																
cg15076811	1p36.22	10940981	*IGR (C1orf127)*	0.057	0.129	6.59E-01	6.24E-01	4	-0.567	0.112	4.46E-07	7.68E-01	4	13.262	2.71E-04	Y
cg27535302	1p34.2	42165766	*GUCA2A*	0.009	0.043	8.35E-01	2.21E-01	13	-0.193	0.023	1.38E-16	3.99E-01	13	16.801	4.15E-05	Y
cg04510459	1p22.1	91582730	*IGR (CDC7)*	0.112	0.114	3.29E-01	9.23E-01	12	0.801	0.115	2.89E-12	3.22E-01	12	18.140	2.05E-05	Y
cg20306265	1p12	117615002	*FAM46C*	-0.058	0.077	4.54E-01	2.70E-01	20	-0.484	0.094	2.81E-07	6.42E-02	20	12.189	4.81E-04	Y
cg18342183	1q31.1	186763783	*IGR (PLA2G4A)*	0.002	0.080	9.80E-01	2.79E-01	18	-0.402	0.049	2.95E-16	1.40E-01	18	18.576	1.63E-05	Y
cg23924526	2p21	45771409	*PRKCE*	0.440	0.251	7.89E-02	6.05E-01	4	-0.431	0.078	3.34E-08	9.57E-02	4	11.018	9.02E-04	N
cg03063511	2p13.1	73930386	*DGUOK*	-0.009	0.047	8.51E-01	5.73E-01	11	-0.247	0.041	2.74E-09	1.09E-01	11	14.239	1.61E-04	Y
cg25325322	2p11.2	84846591	*TRABD2A*	0.126	0.044	4.16E-03	7.89E-01	20	0.265	0.051	1.86E-07	1.39E-01	20	4.291	3.83E-02	N
cg27608154	2q13	111124233	*BCL2L11*	-0.010	0.061	8.76E-01	2.43E-01	11	-0.310	0.056	3.03E-08	2.81E-01	11	13.223	2.76E-04	Y
cg25679431	3p22.3	32147516	*GPD1L*	0.072	0.071	3.14E-01	1.26E-01	9	-0.221	0.043	3.06E-07	1.62E-01	9	12.351	4.41E-04	Y
cg01317029	3q27.1	184345709	*FAM131A*	-0.072	0.172	6.76E-01	4.57E-01	11	0.616	0.122	4.98E-07	1.62E-01	11	10.622	1.12E-03	N
cg02951719	4p16.3	1518424	*IGR (NKX1-1)*	-0.009	0.113	9.37E-01	3.01E-01	20	-0.490	0.090	5.71E-08	1.46E-01	20	11.036	8.94E-04	N
cg05638493	5q31.3	141303800	*SLC25A2*	-0.111	0.095	2.45E-01	9.61E-01	19	-0.611	0.105	6.00E-09	2.78E-01	18	12.395	4.30E-04	Y
cg08631357	5q32	150209647	*SLC6A7*	0.093	0.048	5.47E-02	4.56E-02	20	0.288	0.056	3.18E-07	2.02E-01	20	6.937	8.44E-03	N
cg17781669	5q33.1	150488796	*IGR (NDST1)*	0.025	0.052	6.29E-01	6.02E-02	14	0.274	0.050	5.00E-08	1.30E-01	14	11.993	5.34E-04	Y
cg15538767	5q33.1	150490833	*IGR (NDST1)*	0.050	0.104	6.30E-01	1.13E-01	7	0.552	0.108	3.27E-07	6.20E-01	7	11.223	8.08E-04	N
cg19810457	5q35.3	180247356	*MAPK9*	-0.005	0.079	9.46E-01	5.89E-01	6	-0.346	0.061	1.16E-08	1.18E-01	6	11.619	6.53E-04	N
cg09198601	6p24.3	10318947	*IGR (TFAP2A)*	-0.099	0.128	4.39E-01	2.26E-01	9	-0.742	0.117	2.37E-10	3.87E-01	9	13.746	2.09E-04	Y
cg14466923	6p21.32	33182959	*COL11A2*	-0.096	0.041	1.97E-02	1.22E-01	10	-0.226	0.045	4.59E-07	6.83E-02	10	4.602	3.19E-02	N
cg17430214	6p21.1	41279166	*TREM1*	0.065	0.120	5.88E-01	5.68E-01	10	0.672	0.123	4.60E-08	7.38E-02	10	12.541	3.98E-04	Y
cg00531924	6q21	110958687	*GTF3C6*	-0.148	0.160	3.57E-01	9.34E-01	12	-1.087	0.188	7.54E-09	1.38E-01	12	14.450	1.44E-04	Y
cg24039042	6q27	170271859	*IGR (LOC154449)*	-0.040	0.088	6.51E-01	6.26E-03	4	0.310	0.044	3.10E-12	1.63E-01	4	12.606	3.84E-04	Y
cg01766850	6q27	170271878	*IGR (LOC154449)*	-0.034	0.075	6.51E-01	6.10E-03	4	0.265	0.038	1.59E-12	2.13E-01	4	12.675	3.71E-04	Y
cg05973084	6q27	170271951	*IGR (LOC154449)*	-0.025	0.055	6.50E-01	5.67E-03	4	0.193	0.027	6.15E-13	4.25E-01	4	12.768	3.53E-04	Y
cg09344348	6q27	170271997	*IGR (LOC154449)*	-0.027	0.059	6.50E-01	5.71E-03	4	0.208	0.029	7.58E-13	4.10E-01	4	12.748	3.56E-04	Y
cg18555277	6q27	170272160	*IGR (LOC154449)*	-0.026	0.057	6.50E-01	5.54E-03	4	0.201	0.028	6.84E-13	3.90E-01	4	12.758	3.54E-04	Y
cg01209642	6q27	170272267	*IGR (LOC154449)*	-0.026	0.057	6.50E-01	5.43E-03	4	0.200	0.028	6.67E-13	3.49E-01	4	12.760	3.54E-04	Y
cg21138542	6q27	170272414	*IGR (DLL1)*	-0.035	0.077	6.51E-01	5.86E-03	4	0.271	0.038	1.75E-12	1.97E-01	4	12.665	3.73E-04	Y
cg03899510	6q27	170272602	*IGR (DLL1)*	-0.065	0.145	6.51E-01	1.06E-02	4	0.510	0.079	1.07E-10	9.71E-02	4	12.186	4.81E-04	Y
cg23988375	6q27	170275018	*IGR (DLL1)*	0.085	0.189	6.51E-01	1.21E-02	4	-0.665	0.111	1.97E-09	2.44E-01	4	11.754	6.07E-04	N
cg16108844	6q27	170275085	*IGR (DLL1)*	0.098	0.216	6.51E-01	1.10E-02	3	-0.760	0.133	1.06E-08	1.74E-01	3	11.455	7.13E-04	N
cg23534345	7p22.1	5639075	*RNF216*	-0.244	0.247	3.23E-01	1.36E-02	17	1.189	0.234	3.94E-07	5.89E-01	17	17.719	2.56E-05	Y
cg27103343	7p21.3	10779424	*IGR (NDUFA4)*	-0.063	0.032	4.79E-02	1.09E-01	20	-0.184	0.031	2.96E-09	6.12E-01	20	7.353	6.69E-03	N
cg07227187	7q11.21	64192342	*IGR (ZNF735P)*	-0.068	0.027	1.29E-02	2.91E-03	20	-0.149	0.027	3.66E-08	5.84E-01	20	4.370	3.66E-02	N
cg01391867	7q22.1	99500248	*ZNF394*	0.138	0.150	3.58E-01	2.80E-01	8	0.787	0.136	7.78E-09	4.44E-01	8	10.285	1.34E-03	N
cg23891049	7q33	134679117	*BPGM*	0.019	0.042	6.53E-01	1.05E-01	4	-0.156	0.030	1.70E-07	6.07E-01	4	11.487	7.01E-04	N
cg27057461	7q36.3	158343687	*PTPRN2*	0.140	0.167	4.03E-01	4.81E-01	5	-0.750	0.129	6.02E-09	4.46E-01	5	17.781	2.48E-05	Y
cg04035728	8p21.2	27465950	*CHRNA2*	0.082	0.061	1.79E-01	4.04E-01	20	-0.335	0.062	8.00E-08	5.36E-01	20	22.912	1.70E-06	Y

cg23277389	8q11.21	48921984	*SNAI2*	-0.222	0.100	2.58E-02	1.38E-01	7	-0.601	0.118	3.71E-07	3.16E-01	7	6.010	1.42E-02	N
cg26687619	8q24.22	133904113	*IGR (ST3GAL1)*	0.140	0.172	4.16E-01	3.67E-01	3	-0.716	0.124	8.59E-09	5.88E-01	3	16.247	5.56E-05	Y
cg23402755	10p14	7702004	*ITIH2*	-0.089	0.105	3.97E-01	7.78E-01	12	-0.726	0.099	2.53E-13	5.25E-01	12	19.424	1.05E-05	Y
cg23809813	10q23.31	88457915	*RNLS*	0.012	0.063	8.51E-01	3.54E-01	20	0.388	0.071	4.57E-08	5.26E-01	20	15.713	7.37E-05	Y
cg05399434	10q26.13	121609167	*IGR (FGFR2)*	0.010	0.156	9.48E-01	9.86E-02	20	-0.897	0.119	5.54E-14	5.26E-02	20	21.281	3.97E-06	Y
cg01628053	10q26.13	121609218	*IGR (FGFR2)*	0.011	0.165	9.48E-01	1.02E-01	20	-0.949	0.131	3.52E-13	1.40E-01	20	20.757	5.21E-06	Y
cg24635736	10q26.13	122979534	*PSTK*	-0.023	0.067	7.27E-01	4.75E-01	8	-0.346	0.060	6.12E-09	7.16E-01	8	12.948	3.20E-04	Y
cg16255663	10q26.3	129552735	*MGMT*	0.012	0.040	7.62E-01	9.49E-02	20	-0.204	0.036	8.69E-09	1.05E-01	20	16.444	5.01E-05	Y
cg17686260	10q26.3	129614500	*MGMT*	-0.011	0.038	7.70E-01	2.07E-02	20	-0.213	0.036	4.05E-09	3.03E-01	20	14.636	1.30E-04	Y
cg05741225	10q26.3	132103799	*JAKMIP3*	0.004	0.059	9.52E-01	2.23E-01	6	-0.218	0.039	2.91E-08	2.21E-01	6	9.673	1.87E-03	N
cg10427719	10q26.3	133334898	*CALY*	-0.010	0.061	8.73E-01	3.87E-01	20	-0.262	0.050	1.77E-07	1.21E-01	20	10.179	1.42E-03	N
cg02114346	10q26.3	133378038	*PAOX*	-0.039	0.133	7.70E-01	8.03E-01	11	0.688	0.137	5.26E-07	1.26E-01	11	14.457	1.43E-04	Y
cg11435872	11p11.2	45805924	*SLC35C1*	0.318	0.180	7.70E-02	3.05E-01	20	1.519	0.291	1.88E-07	2.73E-01	20	12.280	4.58E-04	Y
cg05360847	11q13.4	71576873	*IGR (KRTAP5-11)*	0.216	0.215	3.14E-01	6.68E-01	4	-0.942	0.159	3.50E-09	2.02E-01	4	18.758	1.48E-05	Y
cg02850715	11q24.3	130159317	*ST14*	0.204	0.149	1.72E-01	2.84E-01	20	-0.812	0.138	4.14E-09	7.87E-01	20	24.969	5.83E-07	Y
cg21029769	11q24.3	130159620	*ST14*	0.253	0.186	1.73E-01	3.12E-01	20	-1.006	0.184	4.58E-08	9.16E-01	20	23.191	1.47E-06	Y
cg19153300	11q25	134925086	*IGR (LOC283177)*	0.009	0.041	8.20E-01	5.44E-01	20	-0.201	0.033	1.18E-09	1.89E-01	20	15.904	6.66E-05	Y
cg21806090	12p13.33	2088868	*CACNA1C*	-0.123	0.034	2.60E-04	4.56E-01	6	-0.208	0.040	1.66E-07	5.05E-01	6	2.666	1.02E-01	N
cg11915525	12p11.23	27469767	*SMCO2*	0.052	0.094	5.78E-01	4.58E-01	20	-0.502	0.085	3.10E-09	5.37E-01	20	19.218	1.17E-05	Y
cg21922731	12q13.3	56763346	*HSD17B6*	-0.105	0.154	4.94E-01	2.53E-01	9	-0.920	0.167	3.47E-08	8.65E-01	9	12.874	3.33E-04	Y
cg05979241	12q21.2	77326379	*IGR (E2F7)*	0.056	0.069	4.22E-01	8.31E-01	18	0.424	0.068	3.95E-10	3.58E-01	18	14.469	1.42E-04	Y
cg24420742	12q21.2	77328028	*IGR (E2F7)*	-0.058	0.059	3.21E-01	7.77E-01	16	-0.369	0.057	1.24E-10	7.62E-01	16	14.289	1.57E-04	Y
cg20090301	12q21.2	77362321	*IGR (E2F7)*	0.082	0.102	4.22E-01	9.00E-01	9	0.624	0.109	9.45E-09	2.98E-01	9	13.231	2.75E-04	Y
cg20045970	12q24.11	109881214	*GLTP*	0.206	0.185	2.65E-01	4.54E-01	17	-0.704	0.135	1.84E-07	4.11E-01	17	15.800	7.04E-05	Y
cg17632299	13q14.3	52738831	*LECT1*	0.159	0.099	1.10E-01	4.86E-03	20	0.592	0.085	2.67E-12	1.34E-01	20	11.048	8.88E-04	N
cg09557313	13q14.3	52739039	*LECT1*	0.181	0.113	1.11E-01	7.75E-03	20	0.675	0.100	1.37E-11	1.02E-01	20	10.708	1.07E-03	N
cg13822911	13q33.2	106376925	*LINC00460*	0.019	0.109	8.64E-01	4.05E-01	16	-0.649	0.125	2.03E-07	5.71E-02	16	16.167	5.80E-05	Y
cg05099145	16p13.3	1746332	*MAPK8IP3*	0.118	0.284	6.78E-01	1.77E-01	8	-1.613	0.196	2.07E-16	8.07E-01	8	25.120	5.39E-07	Y
cg11220950	16p13.3	1992692	*SYNGR3*	-0.051	0.164	7.54E-01	5.05E-01	17	-1.153	0.223	2.27E-07	5.53E-02	17	15.880	6.75E-05	Y
cg04519296	16p13.3	1993014	*SYNGR3*	0.049	0.167	7.69E-01	3.76E-01	17	-1.037	0.176	3.88E-09	9.73E-02	17	20.008	7.71E-06	Y
cg09397293	16p13.3	2005032	*ZNF598*	-0.052	0.119	6.64E-01	3.32E-01	20	0.688	0.116	3.06E-09	2.85E-01	20	19.765	8.76E-06	Y
cg26804891	16p13.3	2005241	*ZNF598*	-0.026	0.087	7.69E-01	3.38E-01	20	0.539	0.080	1.62E-11	7.60E-02	20	22.849	1.75E-06	Y
cg08576185	16p13.3	2005683	*ZNF598*	-0.056	0.128	6.64E-01	3.16E-01	20	0.740	0.126	4.76E-09	3.59E-01	20	19.524	9.94E-06	Y
cg10470208	16p13.3	2008700	*ZNF598*	-0.032	0.164	8.47E-01	4.74E-01	14	1.112	0.209	1.02E-07	7.58E-02	14	18.529	1.67E-05	Y
cg06998361	16q21	58110599	*IGR (C16orf80)*	-0.073	0.067	2.81E-01	4.37E-02	20	-0.442	0.069	1.35E-10	2.53E-01	20	14.707	1.26E-04	Y
cg15638306	17q21.33	49506292	*NGFR,LOC100288866*	-0.149	0.158	3.46E-01	2.83E-01	15	-1.047	0.206	3.96E-07	3.09E-01	15	11.898	5.62E-04	Y
cg25674938	19p13.3	1360982	*MUM1*	0.009	0.079	9.06E-01	1.26E-01	9	-0.315	0.053	2.97E-09	2.54E-01	8	11.536	6.82E-04	N
cg04698472	19q13.41	51497571	*SIGLEC12*	-0.019	0.031	5.46E-01	3.17E-01	20	-0.170	0.027	1.62E-10	8.24E-02	20	13.657	2.19E-04	Y
cg18795732	19q13.42	55549968	*IGR (SBK3)*	-0.072	0.062	2.44E-01	4.61E-01	8	-0.310	0.051	1.12E-09	3.97E-01	8	8.805	3.00E-03	N
cg02345758	19q13.43	55837753	*NLRP4,NLRP11*	-0.114	0.081	1.56E-01	3.16E-01	5	-0.463	0.070	2.75E-11	7.09E-01	5	10.725	1.06E-03	N
cg23062096	20p13	2747930	*EBF4*	0.028	0.161	8.64E-01	8.72E-01	6	0.912	0.180	3.89E-07	5.32E-01	6	13.430	2.48E-04	Y
cg18120111	21q22.3	42496937	*RSPH1*	0.023	0.070	7.40E-01	1.90E-01	20	-0.400	0.078	2.64E-07	2.44E-01	20	16.304	5.40E-05	Y
cg20460554	21q22.3	42497150	*RSPH1*	0.016	0.048	7.40E-01	1.42E-01	20	-0.273	0.052	1.57E-07	1.21E-01	20	16.636	4.53E-05	Y
cg04903116	22q11.21	21443870	*HIC2*	-0.078	0.072	2.77E-01	2.31E-01	13	-0.356	0.058	9.59E-10	3.16E-01	13	9.011	2.68E-03	N
cg22846809	22q11.21	21646831	*IGR (SDF2L1)*	0.075	0.069	2.77E-01	2.54E-01	13	0.340	0.055	7.80E-10	3.66E-01	13	9.051	2.63E-03	N

Please see the description provided below Table S3.

Table S6: Wald chi-square test to compare probes effects between hypertensive and non-hypertensive subjects for probes that were significant in brain-specific analyses of only one of the two groups

Significant Probes				Hypertensive Subjects					Non-hypertensive Subjects					Comparison		
ProbeID	Chr	ProbePos	Gene	b_{SMR}	SE_{SMR}	P_{SMR}	P_{HEIDI}	N_{HEIDI}	b_{SMR}	SE_{SMR}	P_{SMR}	P_{HEIDI}	N_{HEIDI}	Chisq	P	Sig?
**Plan 4: Hypertensive Subjects**																
cg11750962	1p36.22	11134621	*MTOR*	-0.411	0.075	5.14E-08	9.76E-02	4	0.373	0.291	1.99E-01	8.52E-01	4	6.812	9.06E-03	N
cg12833011	5q35.1	170503531	*KCNIP1*	0.215	0.038	1.91E-08	4.09E-01	5	-0.046	0.076	5.43E-01	7.12E-01	5	9.408	2.16E-03	N
cg23395749	5q35.3	177557245	*IGR (FAM193B)*	-0.791	0.157	5.17E-07	1.01E-01	5	-0.848	0.198	1.83E-05	7.04E-03	5	0.051	8.21E-01	N
**Plan 5: Non-hypertensive Subjects**																
cg09127005	1p34.1	43950718	*IPO13*	-0.041	0.103	6.89E-01	5.47E-01	11	-0.668	0.123	5.13E-08	5.72E-01	11	15.279	9.28E-05	Y
cg11003133	1q23.1	159076601	*AIM2*	-0.077	0.060	2.00E-01	8.94E-01	18	-0.312	0.062	4.62E-07	3.40E-01	18	7.443	6.37E-03	N
cg04198912	1q32.1	205567693	*MFSD4*	0.135	0.091	1.38E-01	3.05E-02	3	0.654	0.123	1.08E-07	7.41E-01	3	11.544	6.80E-04	Y
cg03706720	3q27.1	184513150	*IGR (EPHB3)*	0.190	0.066	3.76E-03	2.09E-01	20	-0.328	0.048	1.05E-11	2.27E-01	20	40.490	1.98E-10	Y
cg06691616	4q31.1	138609944	*IGR (LINC00499)*	-0.027	0.099	7.85E-01	4.62E-01	8	0.522	0.094	2.84E-08	1.65E-01	8	16.114	5.96E-05	Y
cg08631357	5q32	150209647	*SLC6A7*	0.074	0.039	5.45E-02	2.02E-01	18	0.230	0.045	2.76E-07	2.24E-01	18	6.983	8.23E-03	N
cg02769614	6p21.32	32167275	*PPT2-EGFL8,EGFL8*	-0.051	0.110	6.41E-01	6.44E-03	3	0.535	0.089	1.58E-09	2.46E-01	3	17.166	3.42E-05	Y
cg08288383	7q21.3	98051318	*IGR (LMTK2)*	0.057	0.097	5.59E-01	7.22E-01	19	0.658	0.128	2.86E-07	8.87E-02	19	14.012	1.82E-04	Y
cg10308629	7q33	134670051	*BPGM*	0.010	0.128	9.35E-01	1.79E-01	3	-0.520	0.101	2.88E-07	2.57E-01	3	10.584	1.14E-03	Y
cg04388919	7q36.1	149422042	*IGR (ZNF777)*	0.325	0.123	8.01E-03	4.69E-02	10	1.421	0.229	4.97E-10	2.44E-01	10	17.872	2.36E-05	Y
cg07189157	7q36.1	149422316	*IGR (ZNF777)*	0.268	0.099	6.77E-03	9.95E-02	9	1.172	0.166	1.52E-12	4.69E-01	9	21.933	2.82E-06	Y
cg24635736	10q26.13	122979534	*PSTK*	-0.017	0.072	8.11E-01	2.45E-01	3	-0.374	0.072	1.68E-07	8.24E-01	3	12.286	4.56E-04	Y
cg25214093	10q26.13	124447241	*IGR (NKX1-2)*	-0.020	0.054	7.05E-01	6.30E-01	10	0.260	0.047	3.31E-08	5.00E-01	10	15.461	8.42E-05	Y
cg15567360	11q13.4	71611653	*IGR (KRTAP5-11)*	0.156	0.155	3.16E-01	4.87E-01	3	-0.679	0.130	1.67E-07	3.71E-01	3	17.014	3.71E-05	Y
cg03249590	12q13.3	56646415	*ATP5B,SNORD59A*	0.097	0.056	8.22E-02	8.53E-01	3	0.331	0.059	2.00E-08	5.50E-02	3	8.311	3.94E-03	N
cg06679265	12q13.3	56646420	*ATP5B,SNORD59A*	0.105	0.061	8.31E-02	9.59E-01	3	0.359	0.066	4.72E-08	2.41E-01	3	8.053	4.54E-03	N
cg09557313	13q14.3	52739039	*LECT1*	0.135	0.115	2.38E-01	1.78E-01	7	0.547	0.103	1.06E-07	5.67E-01	7	7.138	7.55E-03	N
cg04870949	13q34	113455542	*IGR (DCUN1D2)*	-0.050	0.044	2.52E-01	4.14E-01	18	-0.270	0.053	4.21E-07	1.29E-01	18	10.119	1.47E-03	Y
cg07011318	16p13.3	2004943	*ZNF598*	-0.022	0.050	6.64E-01	3.31E-01	17	0.291	0.046	1.96E-10	1.12E-01	17	21.132	4.29E-06	Y
cg09397293	16p13.3	2005032	*ZNF598*	-0.021	0.049	6.64E-01	3.35E-01	18	0.282	0.044	1.86E-10	1.13E-01	18	21.156	4.23E-06	Y
cg05211189	16p13.3	2005402	*ZNF598*	0.013	0.040	7.54E-01	4.61E-01	18	0.283	0.043	7.47E-11	1.01E-01	18	20.877	4.90E-06	Y
cg08576185	16p13.3	2005683	*ZNF598*	-0.022	0.051	6.64E-01	4.94E-01	16	0.295	0.046	2.00E-10	9.02E-02	16	21.122	4.31E-06	Y
cg06998361	16q21	58110599	*IGR (C16orf80)*	-0.098	0.091	2.77E-01	1.32E-01	11	-0.591	0.109	5.49E-08	6.81E-01	11	12.131	4.96E-04	Y
cg10191399	16q23.3	81693451	*CMIP*	0.034	0.040	3.89E-01	4.55E-01	20	0.246	0.041	1.54E-09	8.86E-02	20	13.820	2.01E-04	Y
cg20002045	21q22.3	42499561	*SLC37A1*	0.027	0.080	7.40E-01	3.21E-01	20	-0.456	0.090	4.49E-07	1.87E-01	20	15.950	6.51E-05	Y
cg01716252	21q22.3	45132690	*ADARB1*	0.104	0.129	4.21E-01	4.86E-01	17	0.827	0.164	4.46E-07	1.32E-01	17	12.007	5.30E-04	Y

Please see the description provided below Table S3.

Table S7: List of Abbreviations

Abbreviation	Explanation
ABCA7	ATP Binding Cassette Subfamily A Member 7
AD	Alzheimer's Disease
ADCY8	Adenylate Cyclase 8
AIM2	Absent in Melanoma 2
ANK1	Ankyrin 1
AP2A2	Adaptor Related Protein Complex 2 Subunit Alpha 2
APOC1	Apolipoprotein C1
APOE	Apolipoprotein E
APP	Amyloid Beta Precursor Protein
Aβ	Amyloid-β
BIN1	Bridging Integrator 1
BPGM	Bisphosphoglycerate Mutase
BRD2	Bromodomain Containing 2
BUG22	Basal Body Upregulated Gene 22
C10orf54	Chromosome 10 Open Reading Frame 54
C16orf80	Chromosome 16 Open Reading Frame 80
CDH23	Cadherin Related 23
CFAP20	Cilia and Flagella Associated Protein 20
CHRNA2	Cholinergic Receptor Nicotinic Alpha 2 Subunit
CHS	Cardiovascular Health Study
CLIC1	Chloride Intracellular Channel 1
CLU	Clusterin
CMIP	C-Maf Inducing Protein
COL11A2	Collagen Type XI Alpha 2 Chain
dbGaP	The Database of Genotypes and Phenotypes
DGUOK	Deoxyguanosine Kinase
DUSP22	Dual Specificity Phosphatase 22
EBF4	Early B Cell Factor Family Member 4
EGFL8	Epidermal Growth Factor-Like Like Domain Multiple 8
eQTL	Expression Quantitative trait Locus
FAM193B	Family with Sequence Similarity 193 Member B
FDR	False Discovery Rate
FHS	Framingham Heart Study
GABA	Gamma-Aminobutyric Acid
GRASP	Genome-Wide Repository of Associations Between SNPs and Phenotypes
GSA	Gene Set Analysis
GSA-SNP2	Gene Set Analysis-Single-Nucleotide Polymorphisms-2
GSEA	Gene Set Enrichment Analysis
GWAS	Genome-Wide Association Study
HEIDI	Heterogeneity in Dependent Instruments
HLA-DPB1	Human Leukocyte Antigen Class II, DP Beta 1
HLA-DQA2	Human Leukocyte Antigen Class II, DQ Alpha 2
HLA-DQB2	Human Leukocyte Antigen Class II, DQ Beta 2
HLA-DRB1	Human Leukocyte Antigen Class II, DR Beta 1
HLA-DRB5	Human Leukocyte Antigen Class II, DR Beta 5
HRS	Health and Retirement Study

HTN	Hypertension
ICD-9	International Classification of Disease codes, Ninth revision
IGR	Inter-Genic Region
IL-18	Interleukin 18
IL-1β	Interleukin 1 Beta
IRB	Institutional Review Board
ITIH2	Inter-Alpha-Trypsin Inhibitor Heavy Chain 2
KEGG	Kyoto Encyclopedia of Genes and Genomes
KRTAP5-11	Keratin Associated Protein 5-11
L1CAM	L1 Cell Adhesion Molecule
LECT1	Leukocyte Cell Derived Chemotaxin 1
LOADFS	Late-Onset Alzheimer's Disease Family Study
LOC100288866	Uncharacterized LOC100288866
LOC154449	Uncharacterized LOC154449
MAPT	Microtubule Associated Protein Tau
MB	Methylene Blue
MHC	Major Histocompatibility Complex
mQTL	Methylation Quantitative trait Locus
MUM1	Melanoma Ubiquitous Mutated Protein 1
MWA	Methylome-Wide Association
NABA	Matrisome Project
NANOS2	Nanos C2HC-Type Zinc Finger 2
NDUFA4	NDUFA4 Mitochondrial Complex Associated
NGFR	Nerve Growth Factor Receptor
NHGRI-EBI GWAS	National Human Genome Research Institute-European Bioinformatics Institute Genome-Wide Association Studies Catalog
NINCDS-ADRDA	National Institute of Neurological and Communicative Disorders and Stroke of the United States-the Alzheimer's Disease and Related Disorders Association
NLRC4	Nucleotide-Binding Oligomerization Domain, Leucine Rich Repeat and Caspase Recruitment Domain Containing 4
NLRP3	Nucleotide-Binding Oligomerization Domain, Leucine Rich Repeat and Pyrin Domain Containing 3
PHLDA1	Pleckstrin Homology Like Domain Family A Member 1
PID	Pathway Interaction Database
PPT2-EGFL8	Palmitoyl-Protein Thioesterase 2-Epidermal Growth Factor-Like Like Domain Multiple 8 Readthrough
PSEN1	Presenilin 1
PSTK	Phosphoseryl-TRNA Kinase
RHBDF2	Rhomboid 5 Homolog 2
RPL13	Ribosomal Protein L13
SIGLEC12	Sialic Acid Binding Immunoglobulin Like Lectin 12
SLC24A4	Solute Carrier Family 24 Member 4
SLC25A2	Solute Carrier Family 25 Member 2
SLC35C1	Solute Carrier Family 35 Member C1
SLC6A7	Solute Carrier Family 6 Member 7
SMR	Summary Data-Based Mendelian Randomization
SNP	Single-Nucleotide Polymorphism
SORBS3	Sorbin And SH3 Domain Containing 3
SORL1	Sortilin Related Receptor 1
ST14	Suppression of Tumorigenicity 14
TCA	Tricarboxylic Acid
TOMM40	Translocase of Outer Mitochondrial Membrane 40
TREM1	Triggering Receptor Expressed on Myeloid Cells 1
TWA	Transcriptome-Wide Association

ZNF394	Zinc Finger Protein 394
ZNF598	Zinc Finger Protein 598
